# Spread of tetracycline resistance genes at a conventional dairy farm

**DOI:** 10.3389/fmicb.2015.00536

**Published:** 2015-05-29

**Authors:** Martina Kyselková, Jiří Jirout, Naděžda Vrchotová, Heike Schmitt, Dana Elhottová

**Affiliations:** ^1^Institute of Soil Biology, Biology Centre of the Czech Academy of SciencesČeské Budějovice, Czech Republic; ^2^Laboratory of Metabolomic and Isotopic Analyses, Global Change Research Centre of the Czech Academy of SciencesČeské Budějovice, Czech Republic; ^3^Institute for Risk Assessment Sciences, Utrecht UniversityUtrecht, Netherlands

**Keywords:** antibiotic resistance spread, animal manure, cattle intestinal microflora, chlortetracycline, dairy cattle, dairy farm, heavy metals, tetracycline resistance genes

## Abstract

The use of antibiotics in animal husbandry contributes to the worldwide problem of increasing antibiotic resistance in animal and human pathogens. Intensive animal production is considered an important source of antibiotic resistance genes released to the environment, while the contribution of smaller farms remains to be evaluated. Here we monitor the spread of tetracycline resistance (TC-r) genes at a middle-size conventional dairy farm, where chlortetracycline (CTC, as intrauterine suppository) is prophylactically used after each calving. Our study has shown that animals at the farm acquired the TC-r genes in their early age (1–2 weeks), likely due to colonization with TC-resistant bacteria from their mothers and/or the farm environment. The relative abundance of the TC-r genes *tet*(W), *tet*(Q), and *tet*(M) in fresh excrements of calves was about 1–2 orders of magnitude higher compared to heifers and dairy cows, possibly due to the presence of antibiotic residues in milk fed to calves. The occurrence and abundance of TC-r genes in fresh excrements of heifers and adult cows remained unaffected by intrauterine CTC applications, with *tet*(O), *tet*(Q), and *tet*(W) representing a “core TC-resistome” of the farm, and *tet*(A), *tet*(M), *tet*(Y), and *tet*(X) occurring occasionally. The genes *tet*(A), *tet*(M), *tet*(Y), and *tet*(X) were shown to be respectively harbored by *Shigella, Lactobacillus* and *Clostridium, Acinetobacter*, and *Wautersiella*. Soil in the farm proximity, as well as field soil to which manure from the farm was applied, was contaminated with TC-r genes occurring in the farm, and some of the TC-r genes persisted in the field over 3 months following the manure application. Concluding, our study shows that antibiotic resistance genes may be a stable part of the intestinal metagenome of cattle even if antibiotics are not used for growth stimulation, and that smaller dairy farms may also contribute to environmental pollution with antibiotic resistance genes.

## Introduction

Agricultural use of antibiotics contributes to the spread of antibiotic resistance genes that may accumulate in human pathogens, thus threatening the treatment of infectious diseases (Smith et al., 2005; Forsberg et al., 2012; Durso and Cook, 2014; Jechalke et al., 2014). Indeed, the same classes of antibiotics as those used in human medicine are administrated to farm animals for disease treatment and prevention, with tetracyclines and beta-lactams being among the most commonly used. In addition, antibiotics are used in many non-EU countries in subtherapeutic doses for animal growth promotion (Chee-Sanford et al., 2009; Chowdhury et al., 2014). Antibiotics entering animal gastrointestinal tracts represent a selection pressure toward antibiotic resistance, and antibiotic resistance genes seem to be a common part of the intestinal metagenome of farm animals (Durso et al., 2011; Lamendella et al., 2011; Wichmann et al., 2014). Antibiotic resistance genes are excreted in manure in the form of intra- and extracellular DNA (Zhang et al., 2013), together with undigested antibiotic residues (Chee-Sanford et al., 2009). Land application of animal waste or simple leaking of waste storage tanks leads to the contamination of soil and water with antibiotic resistance genes, which are now considered as an emerging environmental pollutant (Pruden et al., 2006; Barkovskii and Bridges, 2012; Hong et al., 2013; Zhu et al., 2013). Strikingly, antibiotic resistance genes may be shared between animal, soil and human bacteria via horizontal gene transfer (Kobashi et al., 2007; Forsberg et al., 2012) and the increasing contamination of soil with antibiotic resistance genes may, therefore, contribute to the worldwide problem of the increasing antibiotic resistance and multiresistance.

Most studies with alarming results on the spread of antibiotic resistance genes focused on large pig facilities where antibiotics are used as feed additives (Barkovskii and Bridges, 2012; Hong et al., 2013; Zhu et al., 2013), but the role of dairy farms with more prudent antibiotic use should not be neglected (Santamaría et al., 2011). Our previous results from a medium-size dairy farm in the Czech Republic have indicated that excrements from cattle receiving prophylactically *Metricycline* (intrauterine suppository of chlortetracycline) were a source of tetracycline resistance (TC-r) genes, though they contained no detectable residues of the drug (Kyselková et al., 2015). Some of the TC-r genes could persist in excrement-amended soils for up to several months, as shown in a soil-microcosm experiment (Kyselková et al., 2015). These results have suggested that the prophylactic use of intrauterine chlortetracycline suppositories may not be safe from the point of view of antibiotic resistance spread.

The use of intrauterine suppositories containing tetracyclines for treatment and prevention of puerperal infections in dairy cattle (conforming to EU law) is common in the Czech Republic. This praxis substantially lowers the occurrence of metritis in herds, which otherwise negatively affects the reproductive performance of cattle, thus causing economical lost to farmers (Goshen and Shpigel, 2006; Galvão, 2011). In the Czech Republic, the frequency and administration (prophylaxis vs. treatment only) of tetracycline suppositories varies among farms, but the annual use is estimated to be 310 kg (year 2013, sum of all authorized tetracycline-, chlortetracycline- and oxytetracycline—containing intrauterine veterinary medicinal products, The Institute for State Control of Veterinary Biologicals and Medicines, personal communication). This is a negligible number compared to the amount of tetracyclines used in pigs (e.g., in the form of medicated feed). Suppositories containing tetracyclines for both treatment and prevention of puerperal infections in dairy cattle are authorized also in other countries in EU (e.g., Germany, Austria, Belgium, United Kingdom; http://mri.medagencies.org/Veterinary/product-information), though the information on their annual use is not publicly available. In contrast, these products are authorized only for disease treatment in Canada and not approved in the US at all (American Academy of Veterinary Pharmacology and Therapeutics, http://c.ymcdn.com/sites/www.aavpt.org/resource/resmgr/imported/tetracyclines.pdf; Galvão, 2011).

Compared to the in-feed application of antibiotics, the levels of antibiotic resistance on farms using intrauterine tetracyclines are mostly unknown and reports on antibiotic residues extra uterus are scarce (Hajurka et al., 2003; Kyselková et al., 2015). Hajurka et al. (2003) detected tetracycline residues in blood and milk of cattle for several days following one-time application of 2 or 3 g tetracycline suppositories. Indeed, resorption from uterus and presence of chlortetracycline (CTC) residues in milk and urine of medicated cattle are reported in *Metricycline* medical leaflet. The levels of residues in milk dropped below the allowed maximum residual levels before the fourth day after the administration and the withdrawal period of 4 days is thus considered safe and recommended for this product in the Czech Republic. In contrast, the levels of CTC in feces of medicated cattle are negligible (Kyselková et al., 2015). Despite this fact, tetracycline resistance genes were abundant in feces of dairy cattle receiving prophylactically *Metricycline* (Kyselková et al., 2015). In addition, milk in the withdrawal period is fed to calves at the studied farm (common praxis), which may affect the intestinal resistome of cattle from early age.

Consequently, we focused more specifically on the TC-resistome in a conventional dairy farm with prophylactic *Metricycline* use. The objectives of this work were to assess (i) whether *Metricycline* applications affect the occurrence and abundance of TC-r genes in dairy cattle excrements (ii) possible routes of TC-r gene acquisition and dissemination among animals, and (iii) the dissemination and persistence of TC-r genes in soil to which manure from the farm was applied.

We have started this study with monitoring the occurrence of 12 TC-r genes in excrements from dairy cows at different time points after *Metricycline* treatment, using PCR. Since the first results had not revealed any relationship between the treatment and TC-r gene occurrence, we further assessed possible differences in TC-r gene abundance due to *Metricycline* application, using real-time PCR. 7 TC-r genes that occurred in dairy cow excrements were further monitored (PCR and real-time PCR) in excrements of heifer-calf pairs, in order to see when the TC-resistome was acquired and how the *Metricycline* application in heifers was involved in this process. Finally, we monitored the presence and abundance of 7 TC-r genes in manure and soil from different sites of the farm, and from a field to which the manure was applied, to show the spread of TC-r genes in the environment. Bacterial hosts of certain TC-r genes that occurred at the farm were revealed by PCR screening of isolates from cattle excrements.

## Materials and methods

### The farm

The research was conducted at an anonymous conventional dairy farm in South Bohemia, Czech Republic. The farm has two parts (termed Farm I and Farm II) at 3 km distance. The Farm I holds about 200 adult cows for milk production, most (80%) are of Ceska Straka bread, others are Red Holstein bread or Ceska Straka-Red Holstein and Ceska Straka-Ayshire cross-breads. The Farm I is divided into 4 sections holding 40–50 animals according to their reproduction cycle and milk yield. A birthplace covered with a roof is adjacent to the Farm I.

Typical animal diet at Farm I consists from 18 kg hay, 18 kg maize silage, 7 kg grain mixture supplemented with 750 mg Zn, 150 mg Cu, 675 mg Mn, 30 g Ca, 6 g P, 14 g Na, 14 g Mg, 3 mg Co, and 4 mg Se per day. No antibiotics are added to feed or water. Chlortetracyline (CTC) is used at the farm either locally (*Pederipra Spray*; chlortetracycline hydrochloride, 20 mg/ml skin spray suspension, Laboratorios HIPRA, S.A., Spain) to treat various traumas and other inflammatory processes of the extremities, or in the form of intrauterine suppository (*Metricycline*, 1 g chlortetracycline hydrochloride, KELA Laboratoria n.v., Hoogstraten, Belgium) to prevent post puerperal infections (see below). *Betamox LA* (amoxicillinum trihydricum, 150 mg/ml suspension for injection; Norbrook, Laboratories Ltd., Newry, Northern Ireland) is mainly used to treat postpartum bacterial infections such as sepsis puerperalis and endometritis puerperalis. These antibiotics are used only under veterinary prescription, conforming to EU law.

Cows at the Farm I undergo regular artificial insemination, and give birth at the birthplace. Within few hours after giving birth, cows receive an intrauterine suppository of *Metricycline* (1 g CTC, KELA Laboratoria n.v., Hoogstraten, Belgium) as a prophylaxis of bacterial infections. Each new-borne calf drinks mother's colostrum and within several hours after the birth is placed in an individual calfhouse outside the Farm I. The calfhouses are mobile sheds without floor (the bedding is in direct contact with soil). Calves held in the calfhouses receive mixed milk from cows that are at 0–5 days after calving and cows that receive amoxicillin to treat bacterial infections (i.e., milk from cows within the withdrawal period). Calves at 1 month also receive grain granulate with mineral additives (Zn, Cu, Mn, Ca, P, Na, Mg, Co, and Se, see above) and hay, *ad libitum*, together with milk.

After weaning (at approximately 2–3 months), calves are brought to the Farm II, which holds up to 100 young animals. They stay in individual calfhouses for more 2–3 months and are fed with hay and grain *ad libitum*. At 6 months, calves are taken to the main building of the Farm II, where they are separated according to the sex. The typical heifer diet at the Farm II consists of 18 kg hay, 18 kg silage, and 7 kg grain mixture per day, including mineral additives (see above). At the age of 18 months, bulls are taken to the slaughter and heifers are naturally inseminated by a breeding bull. Pregnant heifers are taken back to the Farm I where they deliver calves. Thereafter they are kept at the Farm I for milk production. The farm has kept this closed system of animal replenishment in place for several decades. The only animal that is purchased on the farm is a breeding bull that is exchanged once per 2 years.

Manure from the Farm I is daily removed and brought to a field at approximately 2 km from the farm. At the year of this study, the manure was piled at the field for 4 months and afterwards spread on the same field. The calfhouses are removed and cleaned after each use. They are put on the same place within 1–6 weeks, after leaving the soil beneath to dry.

### Excrement, milk, manure and soil sampling

Dairy cows (*n* = 21, termed A-W) were sampled at different time points (i.e., 1, 3, 5, 7, 8, 14 or 30 days, or > 3 months) after *Metricycline* application (56 excrement samples altogether). Heifer-calf pairs (*n* = 12) were sampled as follows. Wherever possible, excrements were taken from heifers approximately 4 months before calving (H-t0), after calving just prior to CTC treatment (H-t1), 48 h after CTC treatment (H-t2) and 8 d after CTC treatment (H-t3). Their calves were sampled within the first 2 weeks after birth (C-t1) and at 1–2 months (before weaning; C-t2). At 3 occasions, it was possible to sample calf meconium (within 24 h after birth; C-t0).

Excrement samples from dairy cows and heifers were taken as an anal grab using a sterile glove, in order to avoid any contamination from the ground. Excrement samples from young calves were caught with a sterile glove at the moment of defecation. The samples were transported at 4°C to the laboratory, where they were homogenized and separated into aliquots for DNA isolation (stored at −20°C), bacteria plating (stored at 4°C till the next day), CTC assessment (−20°C) and other chemical analyses (4°C). At 4 occasions, excrements from swallows nesting in the Farm I were taken from either a floor at a corridor that served only to the staff and that was cleaned the night before, or from a railing, using a clean plastic bag, in order to prevent mixing with cow manure. The swallow excrement samples were stored at −20°C prior to DNA isolation.

Milk samples were taken either directly from cows that were at 2 days after *Metricycline* administration (*n* = 8), or from large pots containing mixed milk for calf feeding (*n* = 15). Fresh milk was collected in 50-mL tubes, cooled to 4°C and analyzed for CTC residues within the same day (see below).

Samples of manure at the birthplace (*n* = 3), manure in calfhouses (*n* = 3) and soil between calfhouses (*n* = 3) were taken in autumn 2013. Control soil not impacted by dairy activities (no grazing and no manuring for past 15 years) was sampled on a meadow in a close vicinity of the Farm I in the same year (*n* = 3). The meadow is located on a slope above the farm, so wastes from the farm cannot be washed down to the meadow during rainfalls. Soil under removed calfhouses (*n* = 8), just prior placing a new calfhouse, was taken at several occasions during summer 2014. In addition, we sampled a pile of manure brought during March—July 2014 from the Farm I to a field located approximately at 2 km from the farm (*n* = 5), field soil in the proximity (about 2 m) of the manure pile (*n* = 5) and control soil from the edges of the field (*n* = 5) in June 2014. The manure was spread on the field in July 2014 and we repeated the sampling in the field in October 2014, taking soil just beneath the removed manure pile (*n* = 5), soil in the proximity (about 2 m) from the removed manure pile (*n* = 5) and control soil from the edges of the field (*n* = 5). All environmental samples were sampled with an ethanol-cleaned spade in order to avoid cross-contaminations. Soil was taken from a depth of 10–15 cm, after removing plant roots (if any). Samples were transported in clean plastic bags at 4°C to the laboratory, where they were homogenized and separated into aliquots for DNA isolation (stored at −20°C) and chemical analyses (4°C). Physicochemical properties of soil and manure samples are summarized in Table S1.

### CTC assessment in cow excrements and milk

CTC was extracted from excrements of dairy cows A, B, C, and O at different time points after *Metricycline* application (1, 2, 3, and 8 days; Table 1). CTC was extracted from 3 g excrements with mixture of acetone, 4 M HCl, and deionized water (13:1:6, v/v/v) according to Wang et al. (2010). Extracts were filtered through syringe filter with PVDF membrane (0.22 μm) to remove coagulated proteins and solid particles prior to the analysis. CTC concentration in the extracts was assessed using HP 1050 HPLC instrument (Hewlett Packard, Palo Alto, USA) equipped with Agilent G1315B diode array detector (DAD; Agilent, Santa Clara, USA) on a 3 μm, 150 × 2 mm, Luna C18 (2) column (Phenomenex, Torrance, USA) as described in Kyselková et al. (2013). The limits of detection (LOD) and quantification (LOQ) of the instrument under selected settings were determined based on the Signal-to-Noise Approach as recommended by The International Conference on Harmonization of Technical Requirements for Registration of Pharmaceuticals for Human Use (ICH; www.ich.org), using the signal to noise ratio of 3:1 and 10:1, respectively. The LOD and LOQ were 0.21 and 0.46 μg mL^−1^ extract, i.e., 0.74 and 1.61 μg g^−1^ ww excrements, respectively.

**Table 1:** **Occurrence of TC-r genes in dairy cow excrements**.

**Animal (breed)^a^**	**Number of previous CTC applications**	**Last CTC application**	**Genes for efflux pumps**								**Genes for ribosomal protection**			
			***tet*(A)**	***tet*(B)**	***tet*(C)**	***tet*(L)**	***tet*(V)**	***tet*(Y)**	***tet*(Z)**	***tet*(M)**	***tet*(O)**	***tet*(Q)**	***tet*(W)**	***tet*(X)**
A (C100%)^*^	3	> 3 months	−	−	−	−	−	−	−	+	+	+	+	−
		1 day	−	−	−	−	−	−	−	−	+	+	+	−
		2 days	−	−	−	−	−	−	−	+	+	+	+	−
		3 days	−	−	−	−	−	−	−	−	+	+	+	−
		8 days	−	−	−	−	−	−	−	−	+	+	+	−
B (C100%)^*^	7	> 3 months	+	−	−	−	−	−	−	−	+	+	+	−
		1 day	−	−	−	−	−	−	−	+	+	+	+	−
		2 days	−	−	−	−	−	−	−	−	+	+	+	−
		3 days	−	−	−	−	−	−	−	−	+	+	+	−
		8 days	+	−	−	−	−	−	−	−	+	+	+	−
C (C83%/A17%)^*^	4	> 3 months	−	−	−	−	−	−	−	−	+	+	+	−
		1 day	−	−	−	−	−	−	−	−	+	+	+	−
		2 days	−	−	−	−	−	+	−	−	+	+	+	−
		3 days	−	−	−	−	−	−	−	−	+	+	+	−
		8 days	−	−	−	−	−	−	−	−	+	+	+	+
D (C100%)^*^	3	> 3 months	−	−	−	−	−	−	−	−	+	+	+	−
		1 day	+	−	−	−	−	−	−	−	+	+	+	+
		2 days	−	−	−	−	−	−	−	−	+	+	+	+
		8 days	−	−	−	−	−	−	−	−	+	+	+	+
E (C82%/R18%)^*^	2	> 3 months	−	−	−	−	−	−	−	−	+	+	+	−
		1 day	−	−	−	−	−	−	−	+	+	+	+	−
		2 days	−	−	−	−	−	−	−	+	+	+	+	−
		8 days	−	−	−	−	−	−	−	+	+	+	+	−
F (C83%/R17%)	2	> 3 months	−	−	−	−	−	−	−	−	+	+	+	+
		2 days	−	−	−	−	−	−	−	−	+	+	+	+
		3 days	−	−	−	−	−	−	−	−	+	+	+	+
		8 days	−	−	−	−	−	−	−	+	+	+	+	+
H (C100%)^#^	4	> 3 months	−	−	−	−	−	−	−	−	+	+	+	−
		3 days	−	−	−	−	−	−	−	−	+	+	+	−
		7 days	−	−	−	−	−	−	−	−	+	+	+	−
		3 months	−	−	−	−	−	−	−	−	+	+	+	−
I (C80%/R20%)	7	> 3 months	−	−	−	−	−	−	−	−	+	+	+	−
		3 days	−	−	−	−	−	−	−	−	+	+	+	+
		7 days	−	−	−	−	−	−	−	−	+	+	+	−
		3 months	−	−	−	−	−	−	−	−	+	+	+	−
J (C88%/R12%)	2	30 days	−	−	−	−	−	−	−	−	+	+	+	−
K (C84%/A16%)	6	14 days	−	−	−	−	−	−	−	−	+	+	+	−
L (C100%)	2	> 3 months	−	−	−	−	−	−	−	−	+	+	+	+
		7 days	−	−	−	−	−	−	−	−	+	+	+	+
		14 days	−	−	−	−	−	−	−	−	+	+	+	+
N (C100%)	1	> 3 months	−	−	−	−	−	−	−	−	+	+	+	+
		7 days	−	−	−	−	−	−	−	−	+	+	+	−
		14 days	−	−	−	−	−	−	−	−	+	+	+	+
O (C81%)^*^	7	> 3 months	−	−	−	−	−	+	−	−	+	+	+	−
		1 day	−	−	−	−	−	−	−	−	+	+	+	+
		2 days	−	−	−	−	−	−	−	−	+	+	+	−
		3 days	−	−	−	−	−	−	−	−	+	+	+	+
		8 days	−	−	−	−	−	−	−	−	+	+	+	−
P (C100%)	4	7 days	−	−	−	−	−	−	−	−	+	+	+	−
Q (C100%)	2	5 days	−	−	−	−	−	−	−	−	+	+	+	−
R (C83%/R17%)	2	5 days	−	−	−	−	−	−	−	−	+	+	+	−
S (C82%/R18%)	2	5 days	−	−	−	−	−	−	−	+	+	+	+	−
T (C100%)	5	5 days	−	−	−	−	−	−	−	−	+	+	+	−
U (C100%)	7	5 days	−	−	−	−	−	−	−	−	+	+	+	−
V (C75%/R25%)	5	5 days	−	−	−	−	−	−	−	−	+	+	+	−
W (C100%)	4	5 days	−	−	−	−	−	−	−	−	+	+	+	+

a *C, Ceska straka; R, Red Holstein; A, Ayshire*.

# *The cow received amoxicillin in the year of sampling*.

* *Animals monitored also with qPCR*.

The presence of tetracyclines and beta-lactams in fresh milk samples was assessed with the Twin Sensor KIT034 (Unisensor S. A., Wandre, Belgium) at the National Veterinary Institute in Jihlava, Czech Republic, according to manufacturer's instructions. Twin Sensor is a competitive receptor-based assay in dipstick format for simultaneous detection of beta-lactams and tetracyclines in one single operation (http://www.unisensor.be). This method detects CTC from about 5–7 μg L^−1^ and amoxicillin (used at the farm) from about 3–4 μg L^−1^.

### Bacteria isolation from cattle excrements

Bacteria were isolated from excrements of dairy cows D, E, and F collected at different time points after *Metricycline* application (1, 3, and 8 days; Table 1) by the serial plate dilution method. Excrement suspensions of required dilution were prepared from 2 g (ww) of excrement in sterile tap water. Plates with Tryptic soy agar (for aerobic bacteria), Endo agar (for enteric bacteria) (both Difco™; Becton, Dickinson and Co., USA) and Schaedler agar (for anaerobic bacteria; ready-made plates purchased from DULAB s.r.o., Czech Republic), all supplemented with tetracycline (25 mg L^−1^), were inoculated with 0.1 mL of excrement suspensions diluted respectively to 10^−4^, 10^−3^, and 10^−3^. Tryptic soy agar plates were incubated for 7 days at 28°C and Endo agar plates for 7 days at 37°C. Schaedler plates were placed into Anaerobic jars with Anaerocult® A and Anaerotest® (all Merck KGaA, Germany) to maintain and control anaerobic conditions, and incubated for 14 days at 37°C. Colonies of TC-resistant bacteria were streaked onto corresponding fresh plates supplemented with tetracycline to obtain pure cultures. The pure cultures of TC-resistant isolates were transferred to glycerol stocks (2 full bacteriological loops of bacterial biomass, 700 μL of Tryptic soy broth, 300 μL of 50% glycerol) for long term storage at −80°C.

### DNA isolation a preparation of bacterial lysates

Total DNA was isolated from 0.5 g soil or excrement using the FastDNA SPIN Kit for Soil (MP Biomedicals Europe, Illkirch, France) with an additional step of guanidine thiocyanate washing (Kyselková et al., 2015). A blank sample with 0.5 mL sterile water instead of soil or excrement was included in each batch of samples, in order to control for possible cross-contaminations during the DNA-isolation step. DNA concentration was assessed using NanoDrop 2000 spectrophotometer (Thermo Scientific, Wilmington, DE). All samples were checked for the absence of PCR inhibitors by amplification of 16S rRNA genes (Table S2).

Bacterial lysates for further PCR screening were prepared as follows. One loop of bacterial cells grown on solid media was resuspended in 1 mL sterile water, heated at 95°C for 5 min and frozen at −20°C. Undiluted and 10-times diluted bacterial lysates were verified for the presence of amplifiable DNA by amplification of 16S rRNA genes (Table S2). If no amplification occurred, the heating-freezing cycles were repeated up to 3 times.

### PCR screening of TC-r genes

At first, the presence of the TC-r genes *tet*(A), *tet*(B), *tet*(C), *tet*(L), *tet*(V), *tet*(Y), and *tet*(Z) coding for tetracycline efflux pumps, *tet*(M), *tet*(O), *tet*(Q), and *tet*(W) coding for ribosomal protection proteins, and *tet*(X) coding for NADPH-dependent oxidoreductase was assessed in total DNA from dairy cow excrements (56 samples) using PCR. The genes *tet*(A), *tet*(M), *tet*(O), *tet*(Q), *tet*(W), *tet*(X), and *tet*(Y) were further screened in excrements of heifers (45 samples), calves (26 samples) and swallows (4 samples), in manure and soil, and in bacterial isolates. The PCR reactions (25 μL) contained 1 × KAPA Taq ReadyMix (KAPABiosystems, Wilmington, MA), primers (for concentrations, see Table S2) and DNA template (either 5–50 ng of isolated DNA or 1 μL bacterial lysate, see above). Blank samples (see DNA isolation section), PCR negative controls (sterile water used instead of DNA template) and appropriate positive controls (Table S2) were included. The PCR cycling conditions are in Table S2. Five μL of PCR reaction mixtures were inspected for the presence of specific PCR products in 2% agarose gels stained with ethidium bromide (1 mg L^−1^, 30 min).

### Verification of PCR specificity with cloning-sequencing

Chosen PCR products of *tet*(A), *tet*(M), *tet*(O), *tet*(Q), *tet*(W), *tet*(X), and *tet*(Y) from calf, heifer, dairy cow and swallow excrements, manure and soil samples were purified using GenElute (Sigma-Aldrich Co., St. Louis, MO) or MinElute kit (Qiagen, Hilden, Germany) and cloned to pGEM-T Easy (Promega, Madison, WI) according to manufacturer's protocol. Up to 20 clones per sample were screened by colony PCR for the presence of the cloned genes. The PCR products from 5 to 7 positive clones per sample were sequenced (Sanger dideoxy sequencing) at SEQme s.r.o. (Dobříš, Czech Republic). The sequences were edited in Bioedit 7.0.4.1 software (Hall, 1999), and subjected to nucleotide BLAST (http://blast.st-va.ncbi.nlm.nih.gov/Blast.cgi).

### Accession numbers

Cloned TC-r gene sequences longer than 200 bp were deposited in the GeneBank under accession numbers KP059882—KP059988, and sequences of *rrs* and TC-r genes from bacterial isolates under accession numbers KP331415—KP331428.

### qPCR assessment of TC-r and 16S rRNA genes

The genes *tet*(M), *tet*(Q), *tet*(Y), and *tet*(W) were quantified in excrements from 5 dairy cows and 6 heifer-calf pairs sampled at different time points prior to and after *Metricycline* application, and in environmental samples from Farm I and from field. Serial dilutions of template DNA from different kinds of samples were subjected to 16S rRNA (*rrs*) gene quantification to find the dilution from which the qPCR response was linear (template free of qPCR inhibitors), and these template dilutions were further used for gene quantification. The *rrs* genes were quantified as described in Kyselková et al. (2013), and the *rrs* quantities were used for TC-r gene abundance normalization.

The *tet*(Y) gene was quantified using FastStart Universal SYBR Green Master (ROX; Roche, Basel, Switzerland) and 800 nM primers (Aminov et al., 2002). The thermal cycling included initial denaturation of 10 min at 95°C, followed by 40 cycles of 15 s denaturation at 95°C and 45 s annealing/extension/data acquisition step at 68°C. The genes *tet*(Q), *tet*(M), and *tet*(W) were quantified using primers, TaqMan probes and reaction conditions described in Peak et al. (2007), with KAPA PROBE FAST ABI Prism qPCR Master Mix (KAPABiosystems). All qPCR reactions were performed on StepOne Plus Real-Time PCR system (Applied Biosystems, Foster City, CA). The specificity of qPCR was checked by inspecting PCR product melt curves (for SYBR Green assays) and the presence on a single band after PCR product migration in 3% agarose gel (for all assays).

Limits of detection (LOD) and quantification (LOQ) were assessed for each TC-r gene as follows. Two-fold dilutions of standards containing approximately 10^∧^3 to <1 copies in 6 replicates were analyzed, and LOD and LOQ were calculated from the obtained threshold cycle (*ct*) values with the sofware Genex Enterprise Version 6 (MultiD Analyses AB, Goteborg, Sweden). LOD, determined on 95% probability level for gene detection, were 7, 160, 165, and 5 gene copies per reaction for *tet*(Q), *tet*(M), *tet*(Y), and *tet*(W), respectively. LOQ, determined as the number of gene copies resulting in less than 25% variation coefficient of *ct*, were 65, 561, 674, and 213 for *tet*(Q), *tet*(M), *tet*(Y) and *tet*(W), respectively.

### Data analyses

TC-r gene quantities below LOD were replaced by the corresponding LOD values. TC-r quantities above LOD but below LOQ were replaced by the corresponding LOQ values. Log-values of the ratio of TC-r gene quantity to *rrs* gene quantity were used for statistical analyses. Differences in the relative abundance of TC-r genes between sampling time points for dairy cows and for calves and heifers were assessed with Repeated Measures Analysis of Variance, using GraphPad InStat Version 3.10 (GraphPad Software Inc., San Diego, CA). If a significant difference (at *P* < 0.05) was revealed, pair-wise comparisons were done with Tukey-Kramer Multiple Comparisons Test. Differences in the relative abundance of TC-r genes among farm or field samples were statistically assessed with Kruskal-Wallis Test, followed by Dunn's Multiple Comparisons Test where appropriate (GraphPad InStat 3). Correlations between the relative abundance of TC-r genes and the number of previous *Metricycline* applications in dairy cows, and between the abundance of TC-r genes and heavy metal content in soil and manure (Pearson's correlation coefficient) were calculated with R 2.13.2 (http://www.r-project.org/). Cluster analysis of manure and soil samples, based on the occurrence of TC-r genes, was performed with Primer6 software (Primer-E, Ltd, Plymouth, UK; Clarke and Gorley, 2006).

## Results

### Occurrence of TC-r genes in dairy cow excrements

The occurrence of 12 TC-r genes was monitored in 56 excrement samples from 21 dairy cows at different time points from the last *Metricycline* application (Table 1). The genes *tet*(O), *tet*(Q), and *tet*(W) were found in all excrement samples from all cows. *tet*(X), *tet*(M), *tet*(Y), and *tet*(A) were respectively detected in 30, 16, 5, and 5% excrement samples, but their occurrence was not related to the current CTC treatment, i.e., the genes were found only in some animals under treatment, and also in samples from animals >3 months from the last treatment). The presence of *tet*(A), *tet*(M), *tet*(O), *tet*(Q), *tet*(W), *tet*(X), and *tet*(Y) in excrements of dairy cows was confirmed by cloning-sequencing of chosen PCR products (Table S3). In contrast, *tet*(B), *tet*(C), *tet*(L), *tet*(V), and *tet*(Z) were not detected in any sample.

### Occurrence of TC-r genes in heifer-calf pairs

The occurrence of 7 TC-r genes previously found in dairy cow excrements was monitored in excrements of 12 heifers and their first calves at several time points (Table 2) and subsequently confirmed in selected samples by cloning-sequencing (Table S3). As in the case of dairy cows, *tet*(W) and *tet*(O) were found in all samples, with the exception of one calf at 1–2 months where *tet*(O) was not detected. Strikingly, these two genes were also found in all 3 samples of meconium (Table 2, calf—t0 samples). *tet*(Q) was also frequently (38 out of 45 samples) found in heifer excrements and in all calf excrements but not in meconium. *tet*(A), *tet*(M), *tet*(X), and *tet*(Y) were occasionally detected in excrements of calves (4, 9, 11, and 4 out of 26 samples) and heifers (4, 1, 9, and 4 out of 45 samples), but with no relation to the CTC treatment. In addition, the occurrence of the TC-r genes in calves was not related to their mothers, i.e., the TC-r genes often occurred in calves although they were not detected in their mothers.

**Table 2:** **Occurrence of TC-r genes in heifer-calf pairs**.

**Heifer-calf pair**	**Sample^a^**	**Tetracycline resistance gene**						
		***tet*(A)**	***tet*(M)**	***tet*(O)**	***tet*(Q)**	***tet*(W)**	***tet*(X)**	***tet*(Y)**
Pair 1	Heifer 1 − t0^*^	−	−	+	+	+	−	−
	Heifer 1 – t1^*^	−	−	+	+	+	−	−
	Heifer 1 – t2^*^	−	−	+	+	+	−	−
	Heifer 1 – t3^*^	−	−	+	+	+	−	−
	Calf 1 – t1^*^	−	+	+	+	+	−	−
	Calf 1 – t2^*^	−	−	+	+	+	+	−
Pair 2	Heifer 2 − t0	−	−	+	+	+	−	−
	Heifer 2 – t2	−	−	+	−	+	−	−
	Heifer 2 – t3	−	−	+	+	+	−	−
	Calf 2 – t0	−	+	+	−	+	+	+
	Calf 2 – t1	+	−	+	+	+	−	−
	Calf 2 – t2	−	−	−	+	+	−	−
Pair 3	Heifer 3 − t0	−	−	+	+	+	−	−
	Heifer 3 – t1	−	−	+	+	+	+	−
	Heifer 3 – t2	−	−	+	−	+	−	−
	Heifer 3 – t3	−	−	+	−	+	−	−
	Calf 3 – t1	−	−	+	+	+	−	−
	Calf 3 – t2	−	+	+	+	+	+	+
Pair 4	Heifer 4 − t0^*^	−	−	+	+	+	−	−
	Heifer 4 – t1^*^	−	−	+	+	+	−	−
	Heifer 4 – t2^*^	+	+	+	+	+	−	−
	Heifer 4 – t3^*^	−	−	−	+	+	−	−
	Calf 4 – t1^*^	−	+	+	+	+	−	−
	Calf 4 – t2^*^	−	−	+	+	+	+	−
Pair 5	Heifer 5 − t0	+	−	+	+	+	−	−
	Heifer 5 – t1	−	−	+	+	+	+	−
	Heifer 5 – t2	−	−	+	+	+	+	−
	Heifer 5 – t3	−	−	+	+	+	+	−
	Calf 5 – t0	−	+	+	−	+	−	+
	Calf 5 – t2	−	−	+	+	+	−	−
Pair 6	Heifer 6 − t0^*^	+	−	+	+	+	−	−
	Heifer 6 – t1^*^	−	−	+	+	+	−	−
	Heifer 6 – t2^*^	−	−	+	+	+	−	−
	Heifer 6 – t3^*^	−	−	+	−	+	−	−
	Calf 6 – t1^*^	−	+	+	+	+	+	−
	Calf 6 – t2^*^	−	−	+	+	+	+	−
Pair 7	Heifer 7 - t0^*^	−	−	+	+	+	−	−
	Heifer 7 – t1^*^	−	−	+	+	+	−	−
	Heifer 7 – t2^*^	−	−	+	−	+	−	−
	Heifer 7 – t3^*^	−	−	+	+	+	−	−
	Calf 7 – t0	−	−	+	−	+	−	−
	Calf 7 – t1^*^	−	+	+	+	+	−	−
	Calf 7 – t2^*^	−	−	+	+	+	−	−
Pair 8	Heifer 8 - t0^*^	−	−	+	+	+	−	−
	Heifer 8 − t1^*^	−	−	+	+	+	−	−
	Heifer 8 – t2^*^	+	−	+	+	+	−	−
	Heifer 8 – t3^*^	−	−	+	+	+	−	−
	Calf 8 – t1^*^	−	−	+	+	+	−	−
	Calf 8 – t2^*^	−	−	+	+	+	+	−
Pair 9	Heifer 9 – t1	−	−	+	−	+	−	−
	Heifer 9 – t2	−	−	+	+	+	+	+
	Heifer 9 – t3	−	−	+	+	+	+	−
	Calf 9 – t1	−	−	+	+	+	−	−
	Calf 9 – t2	+	−	+	+	+	+	+
Pair 10	Heifer 10 – t0^*^	−	−	+	+	+	+	+
	Heifer 10 – t1^*^	−	−	+	+	+	+	−
	Heifer 10 – t2^*^	−	−	+	+	+	−	+
	Heifer 10 – t3^*^	−	−	+	+	+	−	−
	Calf 10 – t1^*^	+	+	+	+	+	−	−
	Calf 10 – t2^*^	−	−	+	+	+	+	−
Pair 11	Heifer 11 – t0	−	−	+	+	+	−	+
	Heifer 11 – t1	−	−	+	+	+	−	−
	Heifer 11 – t2	−	−	+	+	+	−	−
	Heifer 11 – t3	−	−	+	+	+	−	−
	Calf 11 – t1	+	+	+	+	+	−	−
	Calf 11 – t2	−	−	+	+	+	+	−
Pair 12	Heifer 12 − t0	−	−	+	+	+	+	−
	Heifer 12 – t1	−	−	+	+	+	−	−
	Heifer 12 – t2	−	−	+	−	+	−	−
	Calf 12 – t1	−	−	+	+	+	−	−
	Calf 12 – t2	−	−	+	+	+	+	−

a *Heifer − t0, heifer 4 months before calving; Heifer − t1, heifer after calving, just prior to CTC application; Heifer − t2, heifer 2d after CTC application; Heifer − t3, heifer 8d after CTC application; Calf − t0, calf within 1 d after the birth (meconium); Calf − t1, calf 1−2 weeks after the birth; Calf − t2, calf 1−2 months after the birth*.

* *Samples monitored also with qPCR*.

### Occurrence of TC-r genes in manure and soil samples

**Figure 1:**
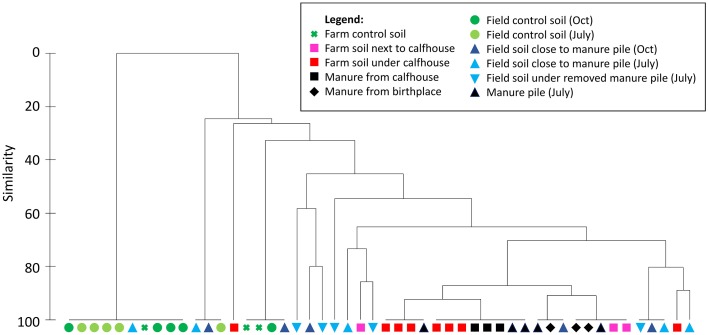
**Similarity among manure and soil samples from the Farm I and field based on the occurrence of TC-r genes**. Bray-Curtis similarities were calculated among individual samples based on PCR results (Table S4) and samples were clustered using group average approach.

Manure sampled on the floor of the birthplace and in the calfhouses contained all 7 TC-r genes that were previously found in animal excrements. These genes leaked to soil under calfhouses as shown at several occasions when calfhouses and manure were removed and the soil beneath was left to dry for at least 1 week (Table S4). This is also illustrated by cluster analysis showing high similarity between manure from calfhouses and soil beneath, based on the occurrence of TC-r genes (Figure 1). Soil just next to the calfhouses displayed an increased contamination with *tet*(A), *tet*(M), *tet*(O), and *tet*(Y), compared to the control meadow soil sampled above the farm, which was not impacted by dairy activities (Table S4, Figure 1). Excrements of swallows found at the farm occasionally contained the TC-r genes (Table S4).

Farm manure piled on a 2-km-distant field during March—July 2014 contained all 7 TC-r genes that were previously found in animal excrements. Cluster analysis has shown that the manure piled on the field, as well as several soil field samples, were highly similar to manure from the farm (calfhouses and birthplace; Figure 1). In detail, field soil sampled in July 2014 at the proximity (about 2 m) of the manure pile was contaminated with all the TC-r genes, while only *tet*(M), *tet*(Y), and *tet*(W) were detected in the soil from the edges of the field (Table S4). Later in July 2014, manure was spread on the field, and the field soil was sampled again in October 2014. At that time, *tet*(A), *tet*(M), *tet*(O), *tet*(W), and *tet*(X) were detected in soil that was beneath the removed manure pile and *tet*(M), *tet*(O), *tet*(W), and *tet*(X) were also found in the 2-m proximity of the removed manure pile, while only *tet*(O) was detected in the control soil from the edge of the field. This result shows that certain TC-r genes persisted in the field soil for at least 3 months after the spread of manure.

The presence of *tet*(A), *tet*(M), *tet*(O), *tet*(W), *tet*(X), and *tet*(Y) in chosen manure, soil and swallow excrement samples was confirmed by cloning-sequencing of chosen PCR products (see Table S3).

### Occurrence of TC-r genes in bacteria isolated from cattle excrements

TC-resistant isolates (*n* = 54) obtained from excrements of 3 dairy cows within 1 week after CTC application were screened for the presence of *tet*(A), *tet*(M), *tet*(O), *tet*(Q), *tet*(W), *tet*(X), and *tet*(Y) in order to identify bacterial hosts of TC-r genes that occurred in the farm (Table 3). Three *Acinetobacter* isolates harbored *tet*(Y), 3 *Shigella* isolates harbored *tet*(A), one *Clostridium* and 2 *Lactobacillus* isolates harbored *tet*(M), and one *Wautersiella* isolate harbored *tet*(X) (the specificity of PCR products verified by sequencing). *tet*(O), *tet*(Q), and *tet*(W) were not detected in any isolate.

**Table 3:** **Bacterial isolates from dairy cattle excrements harboring tetracycline resistance genes**.

**Isolate**	**Closest type strain base on 16S rRNA sequence**	**Growth**	**TC-r gene**
*Acinetobacter* sp. BCCO 40_FK65	*Acinetobacter johnsonii* CIP 64.6	TSA + TET, aerobic	*tet*(Y)
*Acinetobacter* sp. BCCO 40_FK66	*Acinetobacter johnsonii* CIP 64.6	TSA + TET, aerobic	*tet*(Y)
*Acinetobacter* sp. BCCO 40_FK72	*Acinetobacter soli* B1 (T)	TSA + TET, aerobic	*tet*(Y)
*Clostridium* sp. BCCO 40_FK99	*Clostridium tyrobutyricum* ATCC 25755(T)	Schaedler agar, anaerobic	*tet*(M)
*Lactobacillus* sp. BCCO 40_FK89	*Lactobacillus ruminis* NBRC 102161(T)	Schaedler agar, anaerobic	*tet*(M)
*Lactobacillus* sp. BCCO 40_FK97	*Lactobacillus ruminis* NBRC 102161(T)	Schaedler agar, anaerobic	*tet*(M)
*Shigella* sp. BCCO 40_FK53	*Shigella flexneri* ATCC 29903(T)	TSA + TET, aerobic	*tet*(A)
*Shigella* sp. BCCO 40_FK78	*Shigella flexneri* ATCC 29903(T)	TSA + TET, aerobic	*tet*(A)
*Shigella* sp. BCCO 40_FK81	*Shigella flexneri* ATCC 29903(T)	TSA + TET, aerobic	*tet*(A)
*Wautersiella* sp. BCCO 40_FK59	*Wautersiella falsenii* NF 993(T)	TSA + TET, aerobic	*tet*(X)

### Abundance of TC-r genes in cattle excrements

**Figure 2:**
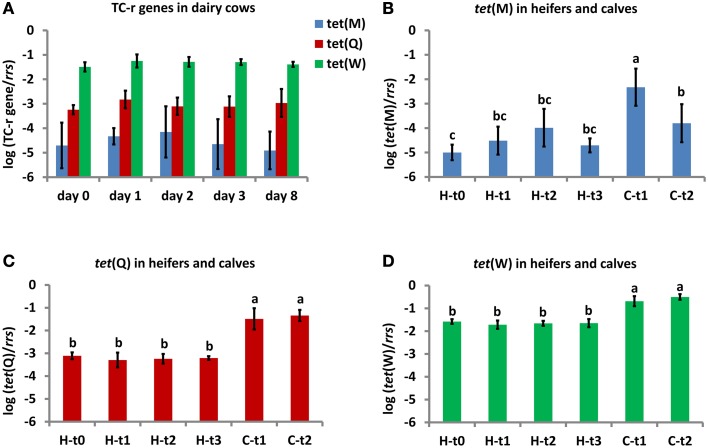
**Relative abundance of TC-r genes in fresh excrements from dairy cows (A), heifers and their calves (B–D)**. TC-r gene abundances were normalized to *rrs* gene copies and expressed on a log-scale (means and standard deviations are shown on bars and error bars). Dairy cows (*n* = 5) were sampled at 0, 1, 2, 3, and 8 days after *Metricycline* suppository administration **(A)**. Heifers (*n* = 6) were sampled at 4 months before the first delivery and at 0, 2, and 8 days after the first *Metricycline* suppository administration (corresponding respectively to H-t0, H-t1, H-t2, and H-t3 in **B**–**D**). Calves (*n* = 6) were sampled at 1–2 weeks and at 1–2 months after birth (corresponding respectively to C-t1 and C-t2). Lower case letters above the error bars indicate statistical differences between time points (*P* < 0.05).

The relative abundance of *tet*(Q) ranged from −2.2 to −3.8 log units related to 16S rRNA gene (*rrs*) copies in dairy cow and heifer excrements, and from −0.7 to −2.1 log units in calf excrements (Figure 2). There was no difference in the *tet*(Q) abundance between the *Metricycline* treatment time points for dairy cows or heifers. Only calf excrements (at both time points) differed significantly from heifer excrements (at all time points) (Figure 2). The Repeated Measures Analysis of Variance further showed that there was a significant effect of individual animal on the *tet*(Q) abundance for dairy cows, while no matching in the *tet*(Q) abundance was found when inspecting individual heifer-calf pairs. The *tet*(Q) abundance in individual dairy cows prior to the actual CTC application was not correlated to the number of previous CTC applications.

The relative abundance of *tet*(W) ranged from −0.9 to −1.9 log units related to *rrs* in dairy cow and heifer excrements, and from −0.3 to −1.1 log units in calf excrements. As in the case of *tet*(Q), there was no difference in the *tet*(W) abundance between the *Metricycline* treatment time points for dairy cows or heifers. Calf excrements (at both time points) differed significantly from heifer excrements (at all time points) (Figure 2). In contrast to *tet*(Q), the Repeated Measures Analysis of Variance did not show any significant effect of individual animals on the *tet*(W) abundance for dairy cows. In addition, no matching in the *tet*(W) abundance was found within individual heifer-calf pairs.

The relative abundance of *tet*(M) varied among the individual animals to a larger extent than the abundance of *tet*(Q) and *tet*(W), as it could be already anticipated from PCR data. It ranged from −2.7 to −5.8 log units related to *rrs* in dairy cow and heifer excrements, and from −1.4 to −4.9 log units in calf excrements. As in the case of *tet*(Q) and *tet*(W), there was no difference in the *tet*(M) abundance between the *Metricycline* treatment time points for dairy cows or heifers. The highest abundance of *tet*(M) was in the excrements of calves up to 2 weeks, which significantly differed from calves at 1–2 months. Heifers at 4 months before the delivery had significantly less *tet*(M) than calves, while in later time points they did not differ from calves at 1–2 months (Figure 2). There was an effect of individual animal on the *tet*(M) abundance in dairy cow excrements, but as in the case of *tet*(Q) this was not correlated to the number of previous *Metricycline* applications. No matching in the *tet*(M) abundance was found within individual heifer-calf pairs.

*tet*(Y) was below LOQ (approximately −5 to −6 log units related to *rrs*) in all cattle excrement samples.

### Abundance of TC-r genes in manure and soil samples

The relative abundance of *tet*(Y) exceeded LOQ values in samples of manure from the birthplace (−3.5 to −4.4 log units related to *rrs*) and calfhouses (−4.0 to −4.1 log units), and soil under calfhouses (−4.0 to −5.0 log units; Figure S1). The relative abundance of *tet*(Q) in manure at the birthplace and in the calfhouses ranged from −3.5 to −3.9 and from −1.5 to −1.9 log units, respectively, while soil samples were mostly at LOQ levels (about −6 log units). The relative abundance of *tet*(M) was above LOQ values not only in samples of manure from the birthplace and calfhouses (about −3 log units in both cases), but also in soil sampled next to and under calfhouses (ranging from −1.7 to −4.1 log units), while it was below LOQ (about −5 log units) in control soil from a meadow above the farm. The relative abundance of *tet*(W) in manure from the birthplace and calfhouses ranged from −1.6 to −1.8 and from −0.5 to −0.9 log units, respectively. The relative abundance of *tet*(W) in soil next to and under calfhouses ranged from −3.9 to −4.0 and from −2.9 to −4.6 log units, respectively, which statistically corresponded to the intermediate position between the manure from calfhouses and control meadow soil (the latter being at the LOQ values).

The manure piled on field, sampled in July 2014, contained considerable levels of TC-r genes, the relative abundance of *tet*(Q), *tet*(W) and *tet*(M) ranging from −2.8 to −4.2, −1.4 to −2.4, and −1.6 to −3.3 log units, respectively (Figure S2). These values were significantly above the background field soil values. The soil in the proximity of manure had intermediate levels of the TC-r genes (relative to the manure pile and the background soil), pointing at the leaking of TC-r genes from the manure to the nearby soil. The abundance of *tet*(Y) was mostly below LOQ in field samples so it was not evaluated statistically.

### CTC content in cow excrements and milk for calf feeding

Residues of CTC (below LOQ 1.61 μg g^−1^ ww) were detected in excrements from 3 dairy cows (B and O) that were 1 day (B, C) or 1–8 days (O) after CTC application. CTC (>5–7 μg L^−1^) was found in milk from CTC-treated cows (6 out of 8 cows at 2 days after CTC application). Since calves receive mixed milk from several cows that are within the withdrawal period, we also checked for the presence of CTC in several mixed fed milk samples. We detected CTC in 4 out of 15 mixed milk samples. In addition, 12 out of 15 mixed milk samples were positive for beta-lactams (amoxicillin).

### Correlations between heavy metal content and TC-r gene abundance

Significant positive correlations (*P* > 0.05, values of Pearsons' *r* > 0.5) between the relative abundance of TC-r genes and heavy metal content in the manure and soil samples (Table S1) from the farm and field were found. The abundances of *tet*(Y) and *tet*(Q) were positively correlated with Zn (r = 0.7 and 0.8, respectively), when considering both manure and soil samples. The correlation between *tet*(Y) and Zn was also significant when considering only soil samples (r = 0.6). The abundance of *tet*(W) was positively correlated with Cu and Zn (r = 0.5 and 0.8, respectively), when considering both manure and soil samples.

## Discussion

This study has shown that TC-r genes were widespread at the studied dairy farm. We could identify a “core resistome,” represented by the genes *tet*(W), *tet*(O), and *tet*(Q), which were found in almost all excrement samples from cattle at different age and antibiotic treatment status. The existence of a common pool of antibiotic resistance genes in a herd of animals, not qualitatively affected by the usage of antibiotics, was reported on several occasions (Looft et al., 2012; Wichmann et al., 2014). It seems that the genes *tet*(W), *tet*(O), and *tet*(Q) are a common occurrence in pig and cattle manure (Peak et al., 2007; Santamaría et al., 2011; Barkovskii and Bridges, 2012; Looft et al., 2012; Zhu et al., 2013; Wichmann et al., 2014). It is possible that these genes are a stable part of genomes of normal animal microflora, or, alternatively, are maintained in animal intestines due to other selection pressures than antibiotics (Baker-Austin et al., 2006), e.g., heavy metals that are used as feed additives at the farm.

Strikingly, the relative abundances of TC-r genes in dairy cattle manure were comparable to the levels reported previously for large pig farms (Zhang et al., 2013). While the TC-r gene abundance correlated with the levels of in-feed administrated antibiotics in several studies (Peak et al., 2007; Looft et al., 2012; Zhu et al., 2013), here the levels of TC-r genes remained unaffected by intrauterine CTC application in cows and heifers. Instead, the abundance of certain genes such as *tet*(Q) and *tet*(M) was animal-dependent, and so was the occurrence of the less frequently detected genes *tet*(A), *tet*(M), *tet*(Y), and *tet*(X). We suppose that the variability in the TC-resistome among individual cows is rather caused by individual differences in the composition of the intestinal microbial community, as shown for example by Durso et al. (2010), than by intrauterine *Metricycline* applications.

The lack of the link between *Metricycline* treatment and TC-r gene occurrence in dairy cow excrements is not that surprising regarding the hardly detectable levels of CTC in animal intestines following the intrauterine bolus application. The startling question is where and when these genes appear in the herd and what are the selection pressures that allow for their maintenance in cattle intestines. To address this question, we monitored the occurrence and abundance of TC-r genes in excrements of heifers prior and after the first CTC treatment and in their calves, as well as several places in the farm.

All TC-r genes previously found in dairy cows were also detected in heifers and calves. We thereby show that calves acquire the TC resistome in their early age, which is documented by the fact that up to 5 different TC-r genes could be found in individual calves at an age of 1–2 weeks. Interestingly, *tet*(O) and *tet*(W) were already detectable in meconium samples from 3 calves, showing possible exchange of TC-r bacteria between mother and fetus. Indeed, the presence of bacteria in human placenta and decidua was shown previously (McDonagh et al., 2004), and a recent metagenomics study revealed the presence of bacterial sequences, including antibiotic resistance genes, in human meconium (Koenig et al., 2011). A high background of antibiotic resistance genes in young animals prior any antibiotic treatment was also shown at a conventional pig farm by Looft et al. (2012).

The occurrence of less frequent TC-r genes was not correlated in the individual heifer-calf pairs, neither was the relative abundance of *tet*(M), *tet*(W), and *tet*(Q) correlated between calves and their mothers. The TC-resistome of calves reflected, therefore, the common TC-resistome of the farm, rather than the resistome of their mothers. Sharing of microflora among dairy cows from the same herd was previously shown on the level of *E. coli* isolates (Sawant et al., 2007). Next to inoculation through the mother (throughout pregnancy, delivery, and colostrum suckling), the farm environment might play an important role in the early calf colonization with TC-r genes. Indeed, the role of the immediate environment on the outcome of the gut colonization was noticed in piglets (Thompson et al., 2008). Our study has shown that one likely route of calf colonization is the manure from the floor of the birthplace, which contained high levels of the monitored TC-r genes. In addition, direct contact with other cows at the birthplace, fed milk, swallows, flies (Rybaříková et al., 2010), aerosols (Hong et al., 2012; Ling et al., 2013) and contaminated soil beneath the calfhouses have to be considered as possible sources of TC-resistant microflora colonizing calves.

Although the TC-r gene pool was shared between calves and older animals, the relative abundance of certain TC-r genes such as *tet*(M), *tet*(Q) and *tet*(W) was 1–2 orders of magnitude higher in excrements of calves in comparison to heifers and dairy cows. It is tempting to suggest that the higher abundance of TC-r genes in calves was due to receiving mixed fed milk from cows that were in the withdrawal period following the application of *Metricycline* or *Betamox* (amoxicillin). Indeed, CTC was detected in several milk samples from individual cows at the second day after *Metricycline* bolus application, though it was often under the detection limit (<5–7 μg L^−1^) in the mixed milk. It has to be noticed that the selection of antibiotic resistance likely occurs at concentrations several times lower than the minimal inhibitory concentrations (referred to as minimal selective concentrations), which could be as little as 30 μg L^−1^ tetracycline (Gullberg et al., 2014), and might be further lowered by the presence of other antibiotics or heavy metals. Indeed, beta-lactams (amoxicillin) were evidenced in the mixed milk samples and these could have contributed to the enrichment of TC-r genes in calf intestines by the mechanism of co-selection. A joint transfer of tetracycline and amoxicillin resistance was shown previously in chicken microflora (van Essen-Zandbergen et al., 2009), and the enrichment of genes conferring resistance to other groups of antibiotics than those administrated was also demonstrated in pigs (Looft et al., 2012). We cannot rule out, however, that the high relative abundance of TC-r genes in calves was due to the variable nature of the developing intestinal community, or the enrichment of certain bacterial populations as a simple response to milk diet (and not necessarily to antibiotic residues). In fact, the intestinal microflora of lactating mammals is likely to be different from that of adults (Palmer et al., 2007; Koenig et al., 2011), and the present study did not allow to distinguish between the effect of milk itself from the effect of antibiotic residues (we could not establish a control group of calves receiving milk from untreated cows).

Contamination of soil with TC-r genes was shown in the proximity of the farm (beneath and between calfhouses), but also at a 2-km distant field on which farm manure was piled and spread. Certain TC-r genes (e.g., *tet*(W) and *tet*(X)) persisted in the field over 3 months following the application of the farm manure, which corroborates our previous results from a soil microcosm experiment (Kyselková et al., 2015). A common occurrence of *tet*(W), *tet*(Q), and *tet*(O) in soil in the proximities of large pig farms (Hong et al., 2013; Zhu et al., 2013) as well as cattle farms (Santamaría et al., 2011) has been previously shown, pointing at the persistence of these genes in the environment. Our study thus shows that not only large pig facilities, but also smaller dairy farms where antibiotics are not used as feed additives might be a source of antibiotic resistance gene contamination for their surroundings. Although the correlations between TC-r genes and heavy metals (mainly Zn) in soil might result from the simultaneous emission of heavy metals and TC-r genes from manure to soil, heavy metals might also represent a selection pressure that helps maintaining the TC-r genes in soil and manure (Baker-Austin et al., 2006).

Bacterial hosts of the “core” TC-r genes *tet*(O), *tet*(Q), and *tet*(W) remained unrevealed in this study, despite of their high relative abundance in cattle excrements. It is likely that these genes occurred in fastidious and/or anaerobic genera such as *Clostridium, Bacteroides, Butyrivibrio*, or *Bifidobacterium*, (http://faculty.washington.edu/marilynr/; January 2014; Roberts, 2005) which are a common part of cattle intestinal microflora (Dowd et al., 2008; Durso et al., 2010) but are difficult to cultivate. In contrast, we have shown for the first time (based on the TC-r gene database at http://faculty.washington.edu/marilynr/; January 2014) the presence of *tet*(Y) in *Acinetobacter* and *tet*(X) in *Wautersiella*. As both of these genera include opportunistic human pathogens, their role in the spread of TC-resistance genes in the farm and its surroundings merits further attention. It is interesting to note that *tet*(Y) was mostly under quantification limits in the excrement (rectal) samples, but its relative abundance increased in manure at the floor of the birthplace and calfhouses. It seems, therefore, that *tet*(Y) hosts (aerobically growing acinetobacters or others) proliferate well outside the animal intestine or that the gene is a subject of horizontal gene transfer to new hosts in the manure.

To sum up, animals in the studied herd harbored TC-r genes from their early age, likely due to colonization with TC-resistant bacteria from both their mothers and the farm environment. Antibiotic residues in fed milk might be a factor temporarily increasing the relative abundance of TC-r genes in calf excrements, while the TC-resistome remains rather stable and unaffected by intrauterine *Metricycline* applications in adult cows. Field application of the manure from the farm increases the contamination of soil with TC-r genes, which persist in soil over several months. Strikingly, this scenario does not much differ from that shown for large pig farms with high antibiotic use (Barkovskii and Bridges, 2012; Hong et al., 2013; Zhu et al., 2013). It remains a question whether antibiotic resistance genes are just a normal part of farm animal microflora or whether they are rare at farms where antibiotics (and heavy metals) have been seldomly used. We suggest that studies on biological farms may answer this question in the future.

### Conflict of interest statement

The authors declare that the research was conducted in the absence of any commercial or financial relationships that could be construed as a potential conflict of interest.
