# Supplementary material for: Spread of tetracycline resistance genes at a conventional dairy farm
Source: Front Microbiol. 2015 May 29;6:536. doi: 10.3389/fmicb.2015.00536 (PMC4448040; doi:10.3389/fmicb.2015.00536)

Supplementary Material

Spread of tetracycline resistance genes at a conventional dairy farm

Martina Kyselková[1*], Jiří Jirout[1], Naděžda Vrchotová[2], Heike Schmitt[3], Dana Elhottová[1]

[1] *Biology Centre of the Czech Academy of Sciences, Institute of Soil Biology, České Budějovice, Czech Republic*

[2] *Global Change Research Centre of the Czech Academy of Sciences*, *Laboratory of Metabolomic and Isotopic Analyses, České Budějovice, Czech Republic*

[3] *Institute for Risk Assessment Sciences, Utrecht University, Utrecht, The Netherlands*

[*]**Correspondence:**

Martina Kyselková, Biology Centre CAS, Institute of Soil Biology, Na Sádkách 7,

370 05 České Budějovice, Czech Republic

E-mail: martinak@upb.cas.cz, Phone: +420 387 775 771, Fax: +420 385 310 133

List of following supplementary tables and figures

Table S1. Physicochemical composition of manure and soil samples

Table S2. PCR detection of tetracycline resistance genes and 16S rRNA (rrs) genes

Table S3. Results of cloning-sequencing of TC-r genes from chosen samples

Table S4. Occurrence of TC-r genes in environmental samples

Figure S1. Relative abundance of TC-r genes in manure and soil samples from Farm I

Figure S2. Relative abundance of TC-r genes in field samples from June 2014

Table S1. Physicochemical composition of manure and soil samples

	texture (USDA)	dry matter (%)	pH	C (%)	N (%)	Cu (mg kg^{-1})	Zn (mg kg^{-1})	Co (mg kg^{-1})	Mn (mg kg^{-1})	Se (mg kg^{-1})
**Samples from Farm I**										
Manure birthplace (n = 3)	n.d.	16.1 ± 2.7	8.5 ± 0.1	39.2 ± 0.6	3.2 ± 0.1	46.9 ± 4.2	244.3 ± 13.3	<5.57	345.7 ± 19.9	<0.56
Manure calfhouses (n = 3)	n.d.	23.3 ± 1.5	7.9 ± 0.1	45.7 ± 1.0	3.6 ± 0.3	63.6 ± 22.1	580.7 ± 149.1	<5.57	426.3 ± 94.1	<0.56
Soil under calfhouses (n = 8)	Silt Loam	80.8 ± 12.8	6.7 ± 0.4	10.8 ± 7.0	0.5 ± 0.5	27.7 ± 14.2	70.9 ± 46.5	<5.57	211.1 ± 33.5	<0.56
Soil next to calfhouses (n = 3)	Silt Loam	86.3 ± 3.7	6.9 ± 0.0	7.9 ± 1.5	0.4 ± 0.1	20.4 ± 4.0	52.9 ± 7.4	<5.57	200.7 ± 23.1	<0.56
Control meadow soils (n = 3)	Silt Loam	88.7 ± 0.6	7.1 ± 0.7	3.5 ± 1.1	0.3 ± 0.0	18.7 ± 2.9	44.0 ± 1.7	6.1 ± 0.2	352.3 ± 18.6	<0.56
**Samples from field (July)**										
Manure pile (n = 5)	n.d.	17.4 ± 3.1	8.6 ± 0.1	35.2 ± 6.7	2.8 ± 0.8	62.8 ± 8.9	301.0 ± 60.6	<5.57	457.0 ± 73.8	<0.56
Soil next to manure pile (n = 5)	Silt Loam	88.2 ± 1.9	6.3 ± 1.2	1.7 ± 0.2	0.2 ± 0.0	39.1 ± 24.2	71.3 ± 1.9	10.5 ± 0.8	715.2 ± 27.1	<0.56

Control field soil (n = 5)	Silt Loam	91.4 ± 2.5	5.0 ± 0.3	2.2 ± 0.5	0.2 ± 0.0	32.6 ± 14.1	75.6 ± 6.9	10.2 ± 0.6	646.4 ± 31.6	<0.56
**Samples from field (October)**										
Soil under manure pile (n = 5)	n.d.	80.7 ± 1.4	5.4 ± 0.2	n.d.	n.d.	n.d.	n.d.	n.d.	n.d.	n.d.
Soil next to manure pile (n = 5)	n.d.	80.3 ± 0.4	5.3 ± 0.2	n.d.	n.d.	n.d.	n.d.	n.d.	n.d.	n.d.
Control field soil (n = 5)	n.d.	79.4 ± 3.0	5.1 ± 0.1	n.d.	n.d.	n.d.	n.d.	n.d.	n.d.	n.d.

Table S2. PCR detection of tetracycline resistance genes and 16S rRNA (*rrs*) genes

Gene	Primers	Primer sequence 5'-3'	Primer concentration [nM]	PCR cycles	Product length [bp]	Positive control
tet(A)	tet(A) (F)	GCTACATCCTGCTTGCCTTC	1000	5 min 95°C, 35 x (30 s 94°C, 30 s 55°C, 30 s 72°C), 5 min 72°C	210	pKJK5 (Bahl et al., 2007)
	tet(A) (R)	CATAGATCGCCGTGAAGAGG	1000			
		(Ng *et al*., 2001)				
tet(B)	tet(B) (F)	TACGTGAATTTATTGCTTCGG	500	4 min 94°C, 35 x (30 s 94°C, 50 s 61°C, 30 s 72°C), 5 min 72°C	206	pRT11 containing 2.7-kb *Hpa*I fragment with *tet*(B) from lambda::Tn10 (Aminov *et al*. 2002)
	tet(B) (R)	ATACAGCATCCAAAGCGCAC	500			
		(Aminov *et al*., 2002)				
tet(C)	tet(C) (F)	GCGGGATATCGTCCATTCCG	100	4 min 94°C, 35 x (20 s 94°C, 30 s 68°C, 50 s 72°C), 5 min 68°C	207	pBR322 (Bolivar *et al*., 1997)
	tet(C) (R)	GCGTAGAGGATCCACAGGACG	100			
		(Aminov *et al*., 2002)				
tet(L)	tet(L) (F)	CATTTGGTCTTATTGGATCG	1000	5 min 94°C, 35 x (30 s 94°C, 30 s 55°C, 1 min 72°C), 3 min 72°C	494	*Staphylococcus aureus* pSTS9-like (Aarestrup *et al*., 2000)
	tet(L) (R)	ATTACACTTCCGATTTCGG	1000			
		(Aarestrup *et al*., 2000)				
tet(M)	tet(M) (F)	GTGGACAAAGGTACAACGAG	500	5 min 95°C, 35 x (30 s 94°C, 30 s 55°C, 1 min 72°C), 5 min 72°C	406	pAT101 containing *tet*(M) from Tn1545 of *Streptococcus pneumoniae* (Martin *et al*., 1986)
	tet(M) (R)	CGGTAAAGTTCGTCACACAC	500			
		(Ng *et al*., 2001)				

tet(O)	tet(O) (F)<br>tet(O) (R)	AACTTAGGCATTCTGGCTCAC<br>TCCCACTGTTCCATATCGTCA<br>(Ng *et al*., 2001)	300<br>300	5 min 95°C, 35 x (30 s 94°C, 30 s 58°C, 30 s 72°C), 5 min 72°C	515	pGEM containing a 2.1 kbp fragment with *tet*(O) from *Butyrivibrio fibrisolvens* (Barbosa *et al*., 1999; Aminov *et al*., 2001)
tet(Q)	tet(Q) (F)<br>tet(Q) (R)	TTATACTTCCTCCGGCATCG<br>ATCGGTTCGAGAATGTCCAC<br>(Ng *et al*., 2001)	1000<br>1000	5 min 95°C, 35 x (30 s 94°C, 30 s 55°C, 1 min 72°C), 5 min 72°C	904	pBT-1containig a 2.5-kb *Sst*I fragment with the *tet*(Q) gene of *Bacteroides thetaiotamicron* (Nikolich *et al*., 1992)
tet(V)	tetV-FW<br>tetV-RV	GCCTACGGTTTCATCCTGGC<br>CGAGACCACCTTCGACAGCG<br>(Kyselková *et al*., 2012)	500<br>500	7 min 95°C, 35 x (1 min 94°C, 15 s 65°C, 30 s 72°C), 5 min 72°C	331	pGEM-T Easy –tetV-2C (351 bp PCR product of *tet*(V) from *Mycobacterium septicum* Site2-2C cloned into pGEM-Teasy) (Kyselková *et al*., 2012)
tet(W)	tet(W) (F)<br>tet(W) (R)	GGGAAATTGTTCGGACAGAC<br>AACGGATACCATCCCTGACA<br>(Call *et al*., 2003)	500<br>500	5 min 95°C, 35 x (30 s 95°C, 30 s 60°C, 50 s 72°C), 10 min 72°C	549	pGEM carrying a 2.4-kb PCR product with the *tet*(W) gene from *Butyrivibrio fibrisolvens* (Barbosa *et al*., 1999)
tet(X)	tetX-1<br>tetX-2	TTAGCCTTACCAATGGGTGT<br>CAAATCTGCTGTTTCACTCG<br>(Bartha *et al*., 2011)	400<br>400	5 min 94°C, 35 x (20 s 94°C, 30 s 56°C, 30s 72°C), 7 min 72°C	242	pHH1107 (Heuer *et al*., 2009)

tet(Y)	tet(Y) (F)<br>tet(Y) (R)	ATTTGTACCGGCAGAGCAAAC<br>GGCGCTGCCGCCATTATGC<br>(Aminov *et al*., 2002)	800<br>800	5 min 94°C, 35 x (30 s 94°C, 30 s 68°C, 30 s 72°C), 5 min 72°C	181	pIE1122 (4.2-kb EcoRV fragment of pIE1120 cloned into pBluescriptKS; Aminov *et al*., 2002*)*
tet(Z)	tet(Z) (F)<br>tet(Z) (R)	CCTTCTCGACCAGGTCGG<br>ACCCACAGCGTGTCCGTC<br>(Aminov *et al*. 2002)	250<br>250	4 min 94°C, 35 x (20 s 94°C, 30 s 61°C, 50 s 72°C), 7 min 68°C	204	pAGHD1 containing *tet*(Z) from pAG1 plasmid of *Corynebacterium glutamicum* (Tauch *et al*., 2000)
rrs	pA<br>pH	AGAGTTTGATCCTGGCTCAG<br>AAGGAGGTGATCCAGCCGCA<br>(Edwards *et al*. 1989)	300<br>300	5min 95°C, 35 x (1 min 94°C, 30 s 61°C, 1,5 min 72°C), 5 min72°C	1500	*Streptomyces rimosus* subsp. *rimosus* DSMZ 40260

References:

Aminov, R. I., Chee-Sanford, J. C., Garrigues, N., Teferedegne, B., Krapac, I.J., White, B.A., et al. (2002). Development, validation, and application of PCR primers for detection of tetracycline efflux genes of Gram-negative bacteria. *Appl. Environ. Microbiol.* 68, 1786–1793.

Aminov, R. I., Garrigues-Jeanjean, N., and Mackie, R. I. (2001). Molecular ecology of tetracycline resistance: development and validation of primers for detection of tetracycline resistance genes encoding ribosomal protection proteins. *Appl. Environ. Microbiol.* 67, 22–32.

Aarestrup, F. M., Agerso, Y., Gerner-Smidt, P., Madsen, M., and Jensen, L. B. (2000). Comparison of antimicrobial resistance phenotypes and resistance genes in *Enterococcus faecalis* and *Enterococcus faecium* from humans in the community, broilers, and pigs in Denmark. *Diagn. Micr. Infec. Dis.* 37, 127–137.

Bahl, M. I., Hansen, L. H., and Sorensen, S. J. (2007) Impact of conjugal transfer on the stability of IncP-1 plasmid pKJK5 in bacterial populations. *FEMS Microbiol. Lett.* 266, 250-256.

Barbosa, T. M., Scott, K. P., and Flint, H. J. (1999). Evidence for recent intergenic transfer of a new tetracycline resistance gene, *tet*(W), isolated from *Butyrivibrio fibrisolvens*, and the occurrence of *tet*(O), in ruminal bacteria. *Environ. Microbiol.* 1, 53–64.

Bartha, N. A., Sóki, J., Urbán, E., and Nagy, E. (2011). Investigation of the prevalence of *tetQ*, *tetX* and *tetX1* genes in *Bacteroides* strains with elevated tigecycline minimum inhibitory concentrations. *Int. J. Antimicrob. Agents*, *38*, 522-525.

Bolivar, F., Rodriguez, R. L., Greene, P. J., Betlach, M. C., Heyneker, H. L., Boyer, H. W., et al. (1977). Construction and characterization of new cloning vehicles. II. A multipurpose cloning system. *Gene* 2, 95–113.

Call, D. R., Bakko, M. K., Krug, M. J., and Roberts, M. C. (2003). Identifying antimicrobial resistance genes with DNA microarrays. *Antimicrob. Agents Chemother.*, 47, 3290-3295.

Edwards, U., Rogall, T., Blöcker, H., Emde, M. and Böttger, E. C. (1989). Isolation and direct complete nucleotide determination of entire genes. Characterization of a gene coding for 16S ribosomal RNA. *Nucleic Acids Res.* 17, 7843–7853.

Heuer, H., Kopmann, C., Binh, C. T., Top, E. M., and Smalla, K. (2009). Spreading antibiotic resistance through spread manure: characteristics of a novel plasmid type with low% G+ C content. *Environ. Microbiol.*, 11, 937-949.

Kyselková, M., Chroňáková, A., Volná, L., Němec, J., Ulmann, V., Scharfen, J., et al. (2012). Tetracycline resistance and presence of tetracycline resistance determinants *tet*(V) and *tap* in rapidly growing mycobacteria from agricultural soils and clinical isolates. *Microbes Environ.* 27, 413-422.

Martin, P., Trieu-Cuot, P., and Courvalin, P. (1986). Nucleotide sequence of the tetM tetracycline resistance determinant of the streptococcal conjugative shuttle transposon Tn1545. *Nucleic Acids Res.* 14, 7047–7058.

Ng, L.-K., Martin, I., Alfa, M., and Mulvey, M. (2001). Multiplex PCR for the detection of tetracycline resistant genes. *Mol. Cell. Probes* 15, 209–215.

Nikolich, M. P., Shoemaker, N. B., and Salyers, A. A. (1992). A Bacteroides tetracycline resistance gene represents a new class of ribosome protection tetracycline resistance. *Antimicrob. Agents Chemother.* 36, 1005–1012.

Tauch, A., Puhler, A., Kalinowski, J., and Thierbach, G. (2000). TetZ, a new tetracycline resistance determinant discovered in gram-positive bacteria, shows high homology to gram-negative regulated efflux systems. *Plasmid* 44, 285–291.

Table S3. Results of cloning-sequencing of TC-r genes from chosen samples

Clone ID	Sample	Amplified gene	Sequence length	BLAST closest hit from cultivated bacteria	Identities with BLAST hit	GeneBank accession # of BLAST hit
KR 138	calf 10 - t1	tet (A)	109	Salmonella enterica subsp. enterica serovar Kentucky strain SRC73, Salmonella Genomic Island 1 variant SGI1-K, CDS 37240-38439 tetA (A) gene for tetracycline efflux protein	109/109 (100%)	AY463797
KR 139	calf 10 - t1	tet (A)	109	Salmonella enterica subsp. enterica serovar Kentucky strain SRC73, Salmonella Genomic Island 1 variant SGI1-K, CDS 37240-38439 tetA (A) gene for tetracycline efflux protein	109/109 (100%)	AY463797
KR 140	calf 10 - t1	tet (A)	109	Salmonella enterica subsp. enterica serovar Kentucky strain SRC73, Salmonella Genomic Island 1 variant SGI1-K, CDS 37240-38439 tetA (A) gene for tetracycline efflux protein	109/109 (100%)	AY463797
KR 141	calf 10 - t1	tet (A)	109	Salmonella enterica subsp. enterica serovar Kentucky strain SRC73, Salmonella Genomic Island 1 variant SGI1-K, CDS 37240-38439 tetA (A) gene for tetracycline efflux protein	109/109 (100%)	AY463797
KR 142	calf 10 - t1	tet (A)	109	Salmonella enterica subsp. enterica serovar Kentucky strain SRC73, Salmonella Genomic Island 1 variant SGI1-K, CDS 37240-38439 tetA (A) gene for tetracycline efflux protein	109/109 (100%)	AY463797
KR 133	heifer 4 - t2	tet (A)	109	Salmonella enterica subsp. enterica serovar Kentucky strain SRC73, Salmonella Genomic Island 1 variant SGI1-K, CDS 37240-38439 tetA (A) gene for tetracycline efflux protein	109/109 (100%)	AY463797
KR 134	heifer 4 - t2	tet (A)	95	Salmonella enterica subsp. enterica serovar Kentucky strain SRC73, Salmonella Genomic Island 1 variant SGI1-K, CDS 37240-38439 tetA (A) gene for tetracycline efflux protein	95/95 (100%)	AY463797
KR 135	heifer 4 - t2	tet (A)	109	Salmonella enterica subsp. enterica serovar Kentucky strain SRC73, Salmonella Genomic Island 1 variant SGI1-K, CDS 37240-38439 tetA (A) gene for tetracycline efflux protein	109/109 (100%)	AY463797
KR 136	heifer 4 - t2	tet (A)	109	Salmonella enterica subsp. enterica serovar Kentucky strain SRC73, Salmonella Genomic Island 1 variant SGI1-K, CDS 37240-38439 tetA (A) gene for tetracycline efflux protein	109/109 (100%)	AY463797
KR 137	heifer 4 - t2	tet (A)	120	Salmonella enterica subsp. enterica serovar Kentucky strain SRC73, Salmonella Genomic Island 1 variant SGI1-K, CDS 37240-38439 tetA (A) gene for tetracycline efflux protein	120/120 (100%)	AY463797
KR 173	dairy cow B - t1	tet (A)	130	Salmonella enterica subsp. enterica serovar Kentucky strain SRC73, Salmonella Genomic Island 1 variant SGI1-K, CDS 37240-38439 tetA (A) gene for tetracycline efflux protein	130/130 (100%)	AY463797
KR 174	dairy cow B - t1	tet (A)	130	Salmonella enterica subsp. enterica serovar Kentucky strain SRC73, Salmonella Genomic Island 1 variant SGI1-K, CDS 37240-38439 tetA (A) gene for tetracycline efflux protein	130/130 (100%)	AY463797
KR 175	dairy cow B - t1	tet (A)	130	Salmonella enterica subsp. enterica serovar Kentucky strain SRC73, Salmonella Genomic Island 1 variant SGI1-K, CDS 37240-38439 tetA (A) gene for tetracycline efflux protein	130/130 (100%)	AY463797
KR 176	dairy cow B - t1	tet (A)	130	Salmonella enterica subsp. enterica serovar Kentucky strain SRC73, Salmonella Genomic Island 1 variant SGI1-K, CDS 37240-38439 tetA (A) gene for tetracycline efflux protein	130/130 (100%)	AY463797
KR 177	dairy cow B - t1	tet (A)	130	Salmonella enterica subsp. enterica serovar Kentucky strain SRC73, Salmonella Genomic Island 1 variant SGI1-K, CDS 37240-38439 tetA (A) gene for tetracycline efflux protein	130/130 (100%)	AY463797
KR 143	manure pile	tet (A)	109	Salmonella enterica subsp. enterica serovar Kentucky strain SRC73, Salmonella Genomic Island 1 variant SGI1-K, CDS 37240-38439 tetA (A) gene for tetracycline efflux protein	109/109 (100%)	AY463797
KR 144	manure pile	tet (A)	109	Salmonella enterica subsp. enterica serovar Kentucky strain SRC73, Salmonella Genomic Island 1 variant SGI1-K, CDS 37240-38439 tetA (A) gene for tetracycline efflux protein	109/109 (100%)	AY463797
KR 145	manure pile	tet (A)	109	Salmonella enterica subsp. enterica serovar Kentucky strain SRC73, Salmonella Genomic Island 1 variant SGI1-K, CDS 37240-38439 tetA (A) gene for tetracycline efflux protein	109/109 (100%)	AY463797
KR 146	manure pile	tet (A)	109	Salmonella enterica subsp. enterica serovar Kentucky strain SRC73, Salmonella Genomic Island 1 variant SGI1-K, CDS 37240-38439 tetA (A) gene for tetracycline efflux protein	109/109 (100%)	AY463797
KR 147	manure pile	tet (A)	109	Salmonella enterica subsp. enterica serovar Kentucky strain SRC73, Salmonella Genomic Island 1 variant SGI1-K, CDS 37240-38439 tetA (A) gene for tetracycline efflux protein	109/109 (100%)	AY463797
KR 21	calf 10 - t1	tet (M)	342	Enterococcus faecalis partial tet (M) gene for tetracycline resistance protein Tet (M), strain ET42	340/341 (99%)	FM202720
KR 22	calf 10 - t1	tet (M)	342	Enterococcus faecalis partial tet (M) gene for tetracycline resistance protein Tet (M), strain ET42	341/341 (100%)	FM202720
KR 23	calf 10 - t1	tet (M)	342	Enterococcus faecalis partial tet (M) gene for tetracycline resistance protein Tet (M), strain ET42	340/341 (99%)	FM202720
KR 24	calf 10 - t1	tet (M)	320	Clostridium difficile strain CD2418 transposon Tn916-like TetM protein (tetM) gene, complete cds	318/320 (99%)	JN846698
KR 25	calf 10 - t1	tet (M)	313	Clostridium difficile strain CD2418 transposon Tn916-like TetM protein (tetM) gene, complete cds	312/313 (99%)	JN846698
KR 26	calf 10 - t1	tet (M)	313	Clostridium difficile strain CD2418 transposon Tn916-like TetM protein (tetM) gene, complete cds	313/313 (100%)	JN846698
KR 99	calf 11 - t1	tet (M)	313	Haemophilus parainfluenzae strain AE-2096513 tet (M)_mega element, CDS 5227-7161: gene tet (M)	313/313 (100%)	KJ545575
KR 100	calf 11 - t1	tet (M)	313	Haemophilus parainfluenzae strain AE-2096513 tet (M)_mega element, CDS 5227-7161: gene tet (M)	313/313 (100%)	KJ545575
KR 101	calf 11 - t1	tet (M)	313	Haemophilus parainfluenzae strain AE-2096513 tet (M)_mega element, CDS 5227-7161: gene tet (M)	313/313 (100%)	KJ545575
KR 102	calf 11 - t1	tet (M)	313	Haemophilus parainfluenzae strain AE-2096513 tet (M)_mega element, CDS 5227-7161: gene tet (M)	313/313 (100%)	KJ545575
KR 103	calf 11 - t1	tet (M)	313	Haemophilus parainfluenzae strain AE-2096513 tet (M)_mega element, CDS 5227-7161: gene tet (M)	313/313 (100%)	KJ545575
KR 27	heifer 4 - t2	tet (M)	313	Clostridium difficile strain CD2418 transposon Tn916-like TetM protein (tetM) gene, complete cds	312/313 (99%)	JN846698
KR 193	heifer 4 - t2	tet (M)	313	Haemophilus parainfluenzae strain AE-2096513 tet (M)_mega element, CDS 5227-7161: gene tet (M)	313/313 (100%)	KJ545575
KR 194	heifer 4 - t2	tet (M)	313	Haemophilus parainfluenzae strain AE-2096513 tet (M)_mega element, CDS 5227-7161: gene tet (M)	311/313 (99%)	KJ545575
KR 195	heifer 4 - t2	tet (M)	313	Haemophilus parainfluenzae strain AE-2096513 tet (M)_mega element, CDS 5227-7161: gene tet (M)	313/313 (100%)	KJ545575
KR 196	heifer 4 - t2	tet (M)	313	Haemophilus parainfluenzae strain AE-2096513 tet (M)_mega element, CDS 5227-7161: gene tet (M)	313/313 (100%)	KJ545575
KR 197	heifer 4 - t2	tet (M)	313	Haemophilus parainfluenzae strain AE-2096513 tet (M)_mega element, CDS 5227-7161: gene tet (M)	313/313 (100%)	KJ545575
KR 188	dairy cow E - t4	tet (M)	313	Haemophilus parainfluenzae strain AE-2096513 tet (M)_mega element, CDS 5227-7161: gene tet (M)	312/313 (99%)	KJ545575
KR 189	dairy cow E - t4	tet (M)	313	Haemophilus parainfluenzae strain AE-2096513 tet (M)_mega element, CDS 5227-7161: gene tet (M)	313/313 (100%)	KJ545575
KR 190	dairy cow E - t4	tet (M)	313	Haemophilus parainfluenzae strain AE-2096513 tet (M)_mega element, CDS 5227-7161: gene tet (M)	312/313 (99%)	KJ545575
KR 191	dairy cow E - t4	tet (M)	313	Haemophilus parainfluenzae strain AE-2096513 tet (M)_mega element, CDS 5227-7161: gene tet (M)	313/313 (100%)	KJ545575
KR 192	dairy cow E - t4	tet (M)	313	Haemophilus parainfluenzae strain AE-2096513 tet (M)_mega element, CDS 5227-7161: gene tet (M)	311/313 (99%)	KJ545575
KR 198	soil under calfhouse	tet (M)	313	Clostridium difficile strain CD2437 transposon Tn916-like TetM protein (tetM) gene, complete cds	313/313 (100%)	JN846700
KR 199	soil under calfhouse	tet (M)	313	Clostridium difficile strain CD2437 transposon Tn916-like TetM protein (tetM) gene, complete cds	313/313 (100%)	JN846700
KR 200	soil under calfhouse	tet (M)	313	Clostridium difficile strain CD2437 transposon Tn916-like TetM protein (tetM) gene, complete cds	313/313 (100%)	JN846700
KR 201	soil under calfhouse	tet (M)	313	Clostridium difficile strain CD2437 transposon Tn916-like TetM protein (tetM) gene, complete cds	312/313 (99%)	JN846700
KR 202	soil under calfhouse	tet (M)	227	Clostridium difficile strain CD2437 transposon Tn916-like TetM protein (tetM) gene, complete cds	227/227 (100%)	JN846700
KR 104	swallow	tet (M)	313	Haemophilus parainfluenzae strain AE-2096513 tet (M)_mega element, CDS 5227-7161: gene tet (M)	313/313 (100%)	KJ545575
KR 105	swallow	tet (M)	313	Haemophilus parainfluenzae strain AE-2096513 tet (M)_mega element, CDS 5227-7161: gene tet (M)	313/313 (100%)	KJ545575
KR 106	swallow	tet (M)	313	Haemophilus parainfluenzae strain AE-2096513 tet (M)_mega element, CDS 5227-7161: gene tet (M)	313/313 (100%)	KJ545575
KR 107	swallow	tet (M)	313	Haemophilus parainfluenzae strain AE-2096513 tet (M)_mega element, CDS 5227-7161: gene tet (M)	312/313 (99%)	KJ545575
KR 108	swallow	tet (M)	313	Haemophilus parainfluenzae strain AE-2096513 tet (M)_mega element, CDS 5227-7161: gene tet (M)	312/313 (99%)	KJ545575
KR 33	calf 1 - t1	tet (O)	426	Campylobacter coli strain Shiraz1 tetracycline resistance gene (tetO) gene, partial cds	426/426 (100%)	JX853721
KR 34	calf 1 - t1	tet (O)	426	Campylobacter coli strain Shiraz1 tetracycline resistance gene (tetO) gene, partial cds	426/426 (100%)	JX853721
KR 35	calf 1 - t1	tet (O)	426	Campylobacter coli strain Shiraz1 tetracycline resistance gene (tetO) gene, partial cds	426/426 (100%)	JX853721
KR 36	calf 1 - t1	tet (O)	426	Campylobacter coli strain Shiraz1 tetracycline resistance gene (tetO) gene, partial cds	426/426 (100%)	JX853721
KR 37	calf 1 - t1	tet (O)	426	Campylobacter coli strain Shiraz1 tetracycline resistance gene (tetO) gene, partial cds	423/426 (99%)	JX853721
KR 28	heifer 1 - t0	tet (O)	426	Campylobacter coli strain Shiraz1 tetracycline resistance gene (tetO) gene, partial cds	422/426 (99%)	JX853721
KR 29	heifer 1 - t0	tet (O)	426	Campylobacter coli strain Shiraz1 tetracycline resistance gene (tetO) gene, partial cds	426/426 (100%)	JX853721
KR 30	heifer 1 - t0	tet (O)	426	Campylobacter coli strain Shiraz1 tetracycline resistance gene (tetO) gene, partial cds	423/426 (99%)	JX853721
KR 31	heifer 1 - t0	tet (O)	426	Campylobacter coli strain Shiraz1 tetracycline resistance gene (tetO) gene, partial cds	423/426 (99%)	JX853721
KR 32	heifer 1 - t0	tet (O)	426	Streptococcus uberis strain IZSSA 647 TetO (tetO) gene, partial cds	420/426 (99%)	KF514877
KR 78	dairy cow B - t4	tet (O)	441	Campylobacter coli strain SH-CCD11C073 tetracycline resistance protein (tetO)	436/441 (99%)	KC876751
KR 79	dairy cow B - t4	tet (O)	441	Campylobacter coli strain SH-CCD11C365 tetracycline resistance protein (tetO)	438/441 (99%)	KC876752
KR 80	dairy cow B - t4	tet (O)	441	Campylobacter coli strain SH-CCD11C073 tetracycline resistance protein (tetO)	440/441 (99%)	KC876751
KR 81	dairy cow B - t4	tet (O)	440	Campylobacter coli strain SH-CCD11C073 tetracycline resistance protein (tetO)	440/441 (99%)	KC876751

KR 82	dairy cow B - t4	tet (O)	299	Streptococcus uberis strain IZSSA 647 TetO (tetO) gene, partial cds	298/299 (99%)	KF514877
KR 158	manure pile	tet (O)	438	Campylobacter coli strain Shiraz1 tetracycline resistance gene (tetO) gene	435/438 (99%)	JX853721
KR 169	manure pile	tet (O)	418	Campylobacter coli strain Shiraz1 tetracycline resistance gene (tetO) gene	417/418 (100%)	JX853721
KR 160	manure pile	tet (O)	418	Campylobacter coli strain Shiraz1 tetracycline resistance gene (tetO) gene	418/418 (100%)	JX853721
KR 161	manure pile	tet (O)	418	Campylobacter coli strain Shiraz1 tetracycline resistance gene (tetO) gene	418/418 (100%)	JX853721
KR 162	manure pile	tet (O)	418	Campylobacter coli strain Shiraz1 tetracycline resistance gene (tetO) gene	418/418 (100%)	JX853721
KR 163	soil close to manure pile	tet (O)	418	Campylobacter coli strain Shiraz1 tetracycline resistance gene (tetO) gene	418/418 (100%)	JX853721
KR 164	soil close to manure pile	tet (O)	418	Campylobacter coli strain Shiraz1 tetracycline resistance gene (tetO) gene	414/418 (99%)	JX853721
KR 165	soil close to manure pile	tet (O)	404	Streptococcus uberis strain IZSSA 647 TetO (tetO) gene, partial cds	403/404 (99%)	KF514877
KR 166	soil close to manure pile	tet (O)	418	Streptococcus uberis strain IZSSA 647 TetO (tetO) gene, partial cds	417/418 (99%)	KF514877
KR 167	soil close to manure pile	tet (O)	418	Streptococcus uberis strain IZSSA 647 TetO (tetO) gene, partial cds	417/418 (99%)	KF514877
KR 168	swallow	tet (O)	418	Campylobacter coli strain Shiraz1 tetracycline resistance gene (tetO) gene	416/418 (99%)	JX853721
KR 169-tetO	swallow	tet (O)	418	Campylobacter coli strain Shiraz1 tetracycline resistance gene (tetO) gene	418/418 (100%)	JX853721
KR 170	swallow	tet (O)	418	Campylobacter coli strain Shiraz1 tetracycline resistance gene (tetO) gene	417/418 (99%)	JX853721
KR 171	swallow	tet (O)	418	Campylobacter coli strain Shiraz1 tetracycline resistance gene (tetO) gene	416/418 (99%)	JX853721
KR 172	swallow	tet (O)	418	Campylobacter coli strain Shiraz1 tetracycline resistance gene (tetO) gene	418/418 (100%)	JX853721
KR 16	calf 1 - t1	tet (Q)	805	Bacteroides fragilis tetA (Q)3 gene	800/805 (99%)	Y08615
KR 17	calf 1 - t1	tet (Q)	805	Bacteroides fragilis tetA (Q)3 gene	793/805 (99%)	Y08615
KR 18	calf 1 - t1	tet (Q)	805	Bacteroides fragilis tetA (Q)3 gene	786/805 (98%)	Y08615
KR 19	calf 1 - t1	tet (Q)	432	Bacteroides fragilis tetA (Q)3 gene	426/432 (99%)	Y08615
KR 20	calf 1 - t1	tet (Q)	805	Bacteroides fragilis tetA (Q)3 gene	790/805 (98%)	Y08615
KR 11	heifer 1 - t0	tet (Q)	805	Bacteroides fragilis tetA (Q)3 gene	805/805 (100%)	Y08615
KR 12	heifer 1 - t0	tet (Q)	805	Bacteroides fragilis tetA (Q)3 gene	805/805 (100%)	Y08615
KR 13	heifer 1 - t0	tet (Q)	805	Bacteroides fragilis tetA (Q)3 gene	805/805 (100%)	Y08615
KR 14	heifer 1 - t0	tet (Q)	805	Bacteroides fragilis tetA (Q)3 gene	805/805 (100%)	Y08615
KR 15	heifer 1 - t0	tet (Q)	805	Bacteroides fragilis tetA (Q)3 gene	797/805 (99%)	Y08615
KR 183	dairy cow B - t4	tet (Q)	585	Bacteroides fragilis partial tetQ gene, strain MN35	585/585 (100%)	FN546887
KR 184	dairy cow B - t4	tet (Q)	585	Bacteroides fragilis partial tetQ gene, strain MN35	585/585 (100%)	FN546887
KR 185	dairy cow B - t4	tet (Q)	539	Bacteroides fragilis partial tetQ gene, strain MN35	538/539 (99%)	FN546887
KR 186	dairy cow B - t4	tet (Q)	585	Bacteroides fragilis partial tetQ gene, strain MN35	584/585 (99%)	FN546887
KR 187	dairy cow B - t4	tet (Q)	488	Bacteroides fragilis partial tetQ gene, strain MN35	473/488 (97%)	FN546887
KR 6	calf 1 - t1	tet (W)	456	Clostridium difficile tetW gene for tetracycline resistance protein, strain EII11	456/456 (100%)	FR838948
KR 7	calf 1 - t1	tet (W)	456	Clostridium difficile tetW gene for tetracycline resistance protein, strain CI7	456/456 (100%)	FR838949
KR 8	calf 1 - t1	tet (W)	456	Clostridium difficile tetW gene for tetracycline resistance protein, strain CI7	455/456 (99%)	FR838949
KR 9	calf 1 - t1	tet (W)	456	Clostridium difficile tetW gene for tetracycline resistance protein, strain EII11	455/456 (99%)	FR838948
KR 10	calf 1 - t1	tet (W)	456	Streptococcus suis tet (W) gene for tetracycline resistance protein, strain 33421	452/456 (99%)	FN356743
KR 1	heifer 1 - t0	tet (W)	456	Clostridium difficile tetW gene for tetracycline resistance protein, strain EII11	456/456 (100%)	FR838948
KR 2	heifer 1 - t0	tet (W)	456	Clostridium difficile tetW gene for tetracycline resistance protein, strain EII11	456/456 (100%)	FR838948
KR 3	heifer 1 - t0	tet (W)	456	Clostridium difficile tetW gene for tetracycline resistance protein, strain EII11	456/456 (100%)	FR838948
KR 4	heifer 1 - t0	tet (W)	456	Bifidobacterium sp. ISO3519 TetW (tetW) gene, complete cds	455/456 (99%)	AF202986
KR 5	heifer 1 - t0	tet (W)	456	Clostridium difficile tetW gene for tetracycline resistance protein, strain EII11	455/456 (99%)	FR838948
KR 178	dairy cow B - t4	tet (W)	475	Clostridium difficile tetW gene for tetracycline resistance protein, strain EII11	473/475 (99%)	FR838948
KR 179	dairy cow B - t4	tet (W)	468	Clostridium difficile tetW gene for tetracycline resistance protein, strain EII11	468/468 (100%)	FR838948
KR 180	dairy cow B - t4	tet (W)	469	Clostridium difficile tetW gene for tetracycline resistance protein, strain EII11	469/469 (100%)	FR838948
KR 181	dairy cow B - t4	tet (W)	469	Clostridium difficile tetW gene for tetracycline resistance protein, strain EII11	469/469 (100%)	FR838948
KR 182	dairy cow B - t4	tet (W)	469	Clostridium difficile tetW gene for tetracycline resistance protein, strain EII11	469/469 (100%)	FR838948
KR 84	manure pile	tet (W)	469	Megasphaera elsdenii strain 29-55 tetracycline resistance protein (tetW) gene	465/469 (99%)	AY485124
KR 85	manure pile	tet (W)	469	Clostridium difficile tetW gene for tetracycline resistance protein, strain EII11	469/469 (100%)	FR838948
KR 86	manure pile	tet (W)	467	Clostridium difficile tetW gene for tetracycline resistance protein, strain EII11	465/467 (99%)	FR838948
KR 87	manure pile	tet (W)	475	Clostridium difficile tetW gene for tetracycline resistance protein, strain EII11	475/475 (100%)	FR838948
KR 88	manure pile	tet (W)	456	Clostridium difficile tetW gene for tetracycline resistance protein, strain CI7	456/456 (100%)	FR838949
KR 89	soil close to manure pile	tet (W)	456	Clostridium difficile tetW gene for tetracycline resistance protein, strain EII11	455/456 (99%)	FR838948
KR 90	soil close to manure pile	tet (W)	439	Clostridium difficile tetW gene for tetracycline resistance protein, strain CI7	429/439 (98%)	FR838949
KR 91	soil close to manure pile	tet (W)	456	Clostridium difficile tetW gene for tetracycline resistance protein, strain EII11	456/456 (100%)	FR838948
KR 92	soil close to manure pile	tet (W)	456	Clostridium difficile tetW gene for tetracycline resistance protein, strain EII11	455/456 (99%)	FR838948
KR 93	soil close to manure pile	tet (W)	455	Clostridium difficile tetW gene for tetracycline resistance protein, strain CI7	455/455 (100%)	FR838948
KR 94	swallow	tet (W)	456	Clostridium difficile tetW gene for tetracycline resistance protein, strain EII11	456/456 (100%)	FR838948
KR 95	swallow	tet (W)	456	Megasphaera elsdenii strain 29-55 tetracycline resistance protein (tetW) gene	454/456 (99%)	AY485124
KR 96	swallow	tet (W)	456	Clostridium difficile tetW gene for tetracycline resistance protein, strain EII11	456/456 (100%)	FR838948
KR 97	swallow	tet (W)	456	Clostridium difficile tetW gene for tetracycline resistance protein, strain CI7	455/456 (99%)	FR838949
KR 98	swallow	tet (W)	456	Clostridium difficile tetW gene for tetracycline resistance protein, strain EII11	456/456 (100%)	FR838948
KR 54	calf 6 - t1	tet (X)	161	Sphingobacterium sp. PM2-P1-29 tetracycline resistance protein (tet (X))	161/161 (100%)	EU864422
KR 55	calf 6 - t1	tet (X)	161	Sphingobacterium sp. PM2-P1-29 tetracycline resistance protein (tet (X))	161/161 (100%)	EU864422
KR 56	calf 6 - t1	tet (X)	161	Sphingobacterium sp. PM2-P1-29 tetracycline resistance protein (tet (X))	161/161 (100%)	EU864422
KR 57	calf 6 - t1	tet (X)	161	Sphingobacterium sp. PM2-P1-29 tetracycline resistance protein (tet (X))	160/161 (99%)	EU864422
KR 58	calf 6 - t1	tet (X)	161	Sphingobacterium sp. PM2-P1-29 tetracycline resistance protein (tet (X))	161/161 (100%)	EU864422
KR 53	heifer 12 - t0	tet (X)	133	Pseudomonas aeruginosa tetX3 gene for tetracycline inactivating enzyme, complete cds	132/133 (99%)	AB097942
KR 64	heifer 12 - t0	tet (X)	338	Wolbachia endosymbiont wPip_Mol of Culex molestus complete genome	35/42 (83%)	HG428761
KR 65	heifer 12 - t0	tet (X)	161	Pseudomonas aeruginosa tetX3 gene for tetracycline inactivating enzyme, complete cds	159/161 (99%)	AB097942

KR 66	heifer 12 - t0	tet (X)	161	Pseudomonas aeruginosa tetX3 gene for tetracycline inactivating enzyme, complete cds	159/161 (99%)	AB097942
KR 67	heifer 12 - t0	tet (X)	161	Pseudomonas aeruginosa tetX3 gene for tetracycline inactivating enzyme, complete cds	158/161 (98%)	AB097942
KR 59	dairy cow D - t3	tet (X)	161	Sphingobacterium sp. PM2-P1-29 tetracycline resistance protein (tet (X))	161/161 (100%)	EU864422
KR 60	dairy cow D - t3	tet (X)	161	Sphingobacterium sp. PM2-P1-29 tetracycline resistance protein (tet (X))	160/161 (99%)	EU864422
KR 61	dairy cow D - t3	tet (X)	161	Sphingobacterium sp. PM2-P1-29 tetracycline resistance protein (tet (X))	161/161 (100%)	EU864422
KR 62	dairy cow D - t3	tet (X)	161	Sphingobacterium sp. PM2-P1-29 tetracycline resistance protein (tet (X))	159/161 (99%)	EU864422
KR 63	dairy cow D - t3	tet (X)	161	Sphingobacterium sp. PM2-P1-29 tetracycline resistance protein (tet (X))	161/161 (100%)	EU864422
KR 148	manure pile	tet (X)	146	Sphingobacterium sp. PM2-P1-29 tetracycline resistance protein (tet (X))	146/146 (100%)	EU864422
KR 149	manure pile	tet (X)	146	Sphingobacterium sp. PM2-P1-29 tetracycline resistance protein (tet (X))	146/146 (100%)	EU864422
KR 150	manure pile	tet (X)	146	Sphingobacterium sp. PM2-P1-29 tetracycline resistance protein (tet (X))	146/146 (100%)	EU864422
KR 151	manure pile	tet (X)	146	Sphingobacterium sp. PM2-P1-29 tetracycline resistance protein (tet (X))	146/146 (100%)	EU864422
KR 152	manure pile	tet (X)	146	Sphingobacterium sp. PM2-P1-29 tetracycline resistance protein (tet (X))	144/146 (99%)	EU864422
KR 153	swallow	tet (X)	146	Sphingobacterium sp. PM2-P1-29 tetracycline resistance protein (tet (X))	146/146 (100%)	EU864422
KR 154	swallow	tet (X)	146	Sphingobacterium sp. PM2-P1-29 tetracycline resistance protein (tet (X))	146/146 (100%)	EU864422
KR 155	swallow	tet (X)	146	Sphingobacterium sp. PM2-P1-29 tetracycline resistance protein (tet (X))	145/146 (99%)	EU864422
KR 156	swallow	tet (X)	146	Sphingobacterium sp. PM2-P1-29 tetracycline resistance protein (tet (X))	146/146 (100%)	EU864422
KR 157	swallow	tet (X)	146	Sphingobacterium sp. PM2-P1-29 tetracycline resistance protein (tet (X))	145/146 (99%)	EU864422
KR 73	calf 9 - t2	tet (Y)	95	Aeromonas bestiarum plasmid pAb5S9, tet (Y) gene for tetracycline resistance efflux protein	95/95 (100%)	EF495198
KR 74	calf 9 - t2	tet (Y)	108	No significant similarity found		
KR 75	calf 9 - t2	tet (Y)	111	Aeromonas bestiarum plasmid pAb5S9, tet (Y) gene for tetracycline resistance efflux protein	111/111 (100%)	EF495198
KR 76	calf 9 - t2	tet (Y)	111	Aeromonas bestiarum plasmid pAb5S9, tet (Y) gene for tetracycline resistance efflux protein	111/111 (100%)	EF495198
KR 77	calf 9 - t2	tet (Y)	111	Aeromonas bestiarum plasmid pAb5S9, tet (Y) gene for tetracycline resistance efflux protein	111/111 (100%)	EF495198
KR 72	heifer 10 - t0	tet (Y)	103	Aeromonas bestiarum plasmid pAb5S9, tet (Y) gene for tetracycline resistance efflux protein	103/103 (100%)	EF495198
KR 129	heifer 10 - t0	tet (Y)	93	Aeromonas bestiarum plasmid pAb5S9, tet (Y) gene for tetracycline resistance efflux protein	92/93 (99%)	EF495198
KR 130	heifer 10 - t0	tet (Y)	93	Aeromonas bestiarum plasmid pAb5S9, tet (Y) gene for tetracycline resistance efflux protein	93/93 (100%)	EF495198
KR 131	heifer 10 - t0	tet (Y)	93	Aeromonas bestiarum plasmid pAb5S9, tet (Y) gene for tetracycline resistance efflux protein	93/93 (100%)	EF495198
KR 132	heifer 10 - t0	tet (Y)	93	Aeromonas bestiarum plasmid pAb5S9, tet (Y) gene for tetracycline resistance efflux protein	93/93 (100%)	EF495198
KR 69	dairy cow O - t0	tet (Y)	94	Aeromonas bestiarum plasmid pAb5S9, tet (Y) gene for tetracycline resistance efflux protein	94/94 (100%)	EF495198
KR 71	dairy cow O - t0	tet (Y)	114	Aeromonas bestiarum plasmid pAb5S9, tet (Y) gene for tetracycline resistance efflux protein	114/114 (100%)	EF495198
KR 109	dairy cow O - t0	tet (Y)	93	Aeromonas bestiarum plasmid pAb5S9, tet (Y) gene for tetracycline resistance efflux protein	93/93 (100%)	EF495198
KR 110	dairy cow O - t0	tet (Y)	93	Aeromonas bestiarum plasmid pAb5S9, tet (Y) gene for tetracycline resistance efflux protein	93/93 (100%)	EF495198
KR 111	dairy cow O - t0	tet (Y)	93	Aeromonas bestiarum plasmid pAb5S9, tet (Y) gene for tetracycline resistance efflux protein	93/93 (100%)	EF495198
KR 112	dairy cow O - t0	tet (Y)	93	Aeromonas bestiarum plasmid pAb5S9, tet (Y) gene for tetracycline resistance efflux protein	93/93 (100%)	EF495198
KR 113	dairy cow O - t0	tet (Y)	93	Aeromonas bestiarum plasmid pAb5S9, tet (Y) gene for tetracycline resistance efflux protein	93/93 (100%)	EF495198
KR 124	manure calfhouse	tet (Y)	92	Aeromonas bestiarum plasmid pAb5S9, tet (Y) gene for tetracycline resistance efflux protein	91/92 (99%)	EF495198
KR 125	manure calfhouse	tet (Y)	93	Aeromonas bestiarum plasmid pAb5S9, tet (Y) gene for tetracycline resistance efflux protein	93/93 (100%)	EF495198
KR 126	manure calfhouse	tet (Y)	93	Aeromonas bestiarum plasmid pAb5S9, tet (Y) gene for tetracycline resistance efflux protein	92/93 (99%)	EF495198
KR 127	manure calfhouse	tet (Y)	93	Aeromonas bestiarum plasmid pAb5S9, tet (Y) gene for tetracycline resistance efflux protein	93/93 (100%)	EF495198
KR 128	manure calfhouse	tet (Y)	93	Aeromonas bestiarum plasmid pAb5S9, tet (Y) gene for tetracycline resistance efflux protein	92/93 (99%)	EF495198
KR 118	manure pile	tet (Y)	93	Aeromonas bestiarum plasmid pAb5S9, tet (Y) gene for tetracycline resistance efflux protein	93/93 (100%)	EF495198
KR 119	manure pile	tet (Y)	93	Aeromonas bestiarum plasmid pAb5S9, tet (Y) gene for tetracycline resistance efflux protein	93/93 (100%)	EF495198
KR 121	manure pile	tet (Y)	93	Aeromonas bestiarum plasmid pAb5S9, tet (Y) gene for tetracycline resistance efflux protein	92/93 (99%)	EF495198
KR 122	manure pile	tet (Y)	93	Aeromonas bestiarum plasmid pAb5S9, tet (Y) gene for tetracycline resistance efflux protein	93/93 (100%)	EF495198
KR 123	manure pile	tet (Y)	93	Aeromonas bestiarum plasmid pAb5S9, tet (Y) gene for tetracycline resistance efflux protein	93/93 (100%)	EF495198
KR 114	swallow	tet (Y)	93	Aeromonas bestiarum plasmid pAb5S9, tet (Y) gene for tetracycline resistance efflux protein	93/93 (100%)	EF495198
KR 115	swallow	tet (Y)	93	Aeromonas bestiarum plasmid pAb5S9, tet (Y) gene for tetracycline resistance efflux protein	93/93 (100%)	EF495198
KR 116	swallow	tet (Y)	93	Aeromonas bestiarum plasmid pAb5S9, tet (Y) gene for tetracycline resistance efflux protein	93/93 (100%)	EF495198
KR 117	swallow	tet (Y)	93	Aeromonas bestiarum plasmid pAb5S9, tet (Y) gene for tetracycline resistance efflux protein	92/93 (99%)	EF495198
KR 120	swallow	tet (Y)	93	Aeromonas bestiarum plasmid pAb5S9, tet (Y) gene for tetracycline resistance efflux protein	93/93 (100%)	EF495198

Table S4. Occurrence of TC-r genes in environmental samples

The TC-r genes were detected (+) or not (-) by PCR and qPCR in 3-8 replicate samples of manure, soil and swallow excrements from the farm or from a 2 km-distant field on which manure was piled and later spread.

			***tet*(A)**	***tet*(M)**	***tet*(O)**	***tet*(Q)**	***tet*(W)**	***tet*(X)**	**tet(Y)**
Farm	Farm control soil (meadow)	PCR	- - -	- - -	- - -	- - -	+ + -	- - -	- - -
		qPCR	nd	- + -	nd	+ - -	+ + +	nd	- - -
	Soil next to calfhouse	PCR	+ + +	- - -	+ - +	- - -	+ + +	+ + +	+ + +
		qPCR	nd	+ + +	nd	+ + +	+ + +	nd	+ + +
	Soil under calfhouse	PCR	- + + + + + + +	- + + + + + + +	- + + + + + + +	- - - + + - + -	- + + + + + + +	+ + + + + + + +	- - + + + + + +
		qPCR	nd	+ + + + +	nd	- - + + +	+ + + + +	nd	+ + + + +
	Manure from calfhouses	PCR	+ + +	+ + +	+ + +	+ + +	+ + +	+ + +	+ + +
		qPCR	nd	+ + +	nd	+ + +	+ + +	nd	+ + +
	Manure from the birthplace	PCR	+ + +	- - -	+ + +	+ - -	+ + +	+ + +	+ + +

		qPCR	nd	+ + +	nd	+ + +	+ + +	nd	+ + +
	Swallow excrements	PCR	+ - - -	+ - - -	+ + - -	- - - -	+ + - -	+ - - -	+ - - -
		qPCR	nd	+ + + -	nd	+ + - -	+ + - -	nd	+ - - -
Field (July 2014)	Field control soil	PCR	- - - - -	- - - - -	- - - - -	- - - - -	- - - - +	- - - - -	- - - - -
		qPCR	nd	+ - + + +	nd	- - - - -	+ + + + +	nd	- - + + -
	Field soil close to manure pile	PCR	- + + - -	- - - - -	+ + + + -	- + - - -	+ + + - +	+ + - - -	- + - - -
		qPCR	nd	+ + + + +	nd	+ + + + +	+ + + + +	nd	+ + + + +
	Manure pile	PCR	+ + + + +	+ + + - -	+ + + + +	- + + + -	+ + + + +	+ + + + +	+ + + + +
		qPCR	nd	+ + + + +	nd	+ + + + +	+ + + + +	nd	+ + + + +
Field (October 2014)	Field control soil	PCR	- - - - -	- - - - -	- - + - -	- - - - -	- - - - -	- - - - -	- - - - -
		qPCR	nd	nd	nd	nd	nd	nd	nd

Field soil close to manure pile	PCR	- - - - -	- - + - -	- + + + -	- - - - -	+ + + - -	+ + + - -	- - - - -
	qPCR	nd	nd	nd	nd	nd	nd	nd
Field soil under the removed manure pile	PCR	+ - + + +	- - + - -	- + - + -	- - - - -	+ + + - -	+ + - - -	- - - - -
	qPCR	nd	nd	nd	nd	nd	nd	nd

nd – not done

Figure S1. Relative abundance of TC-r genes in manure and soil samples from Farm I.

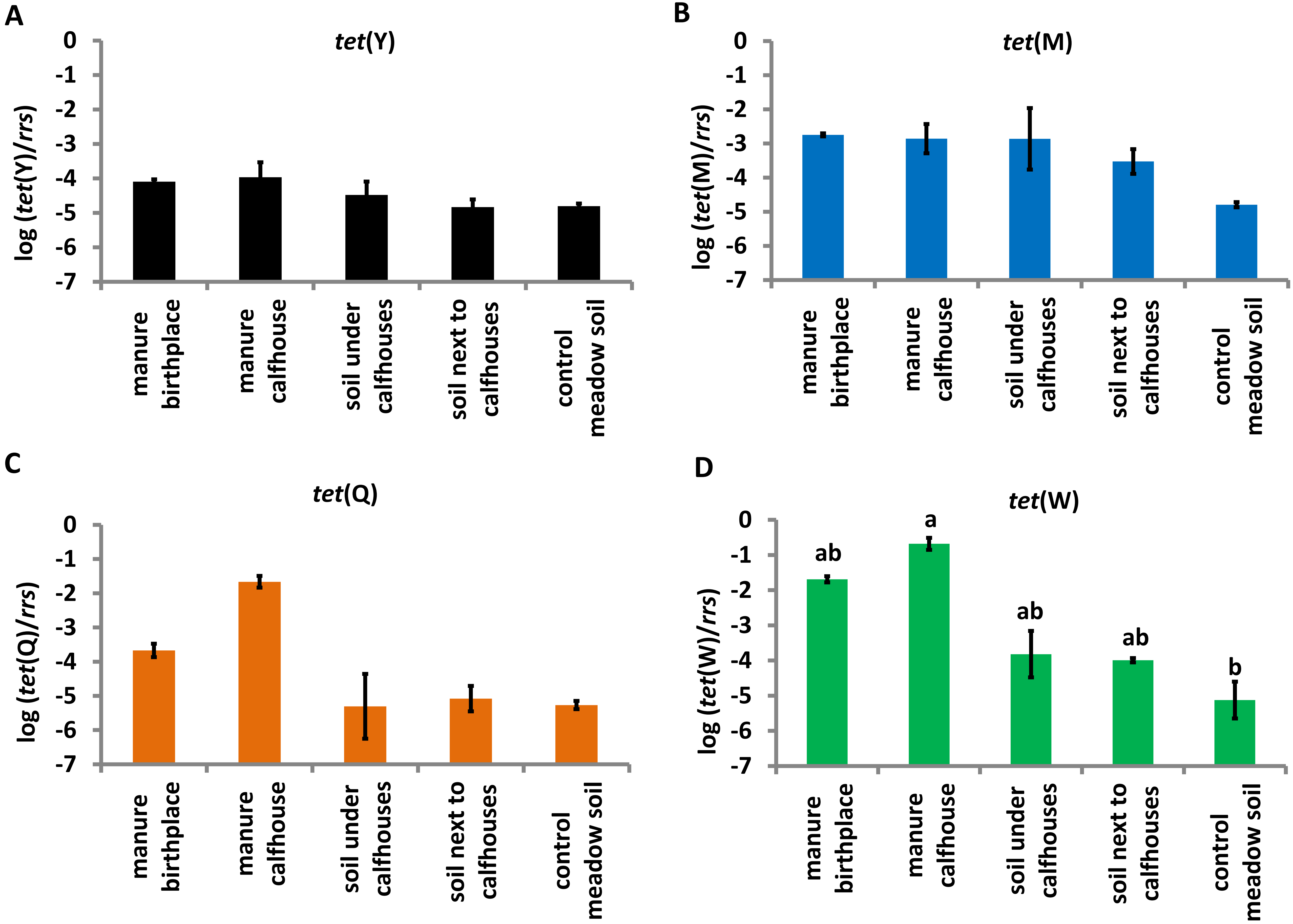

Figure S2. Relative abundance of TC-r genes in field samples from June 2014.

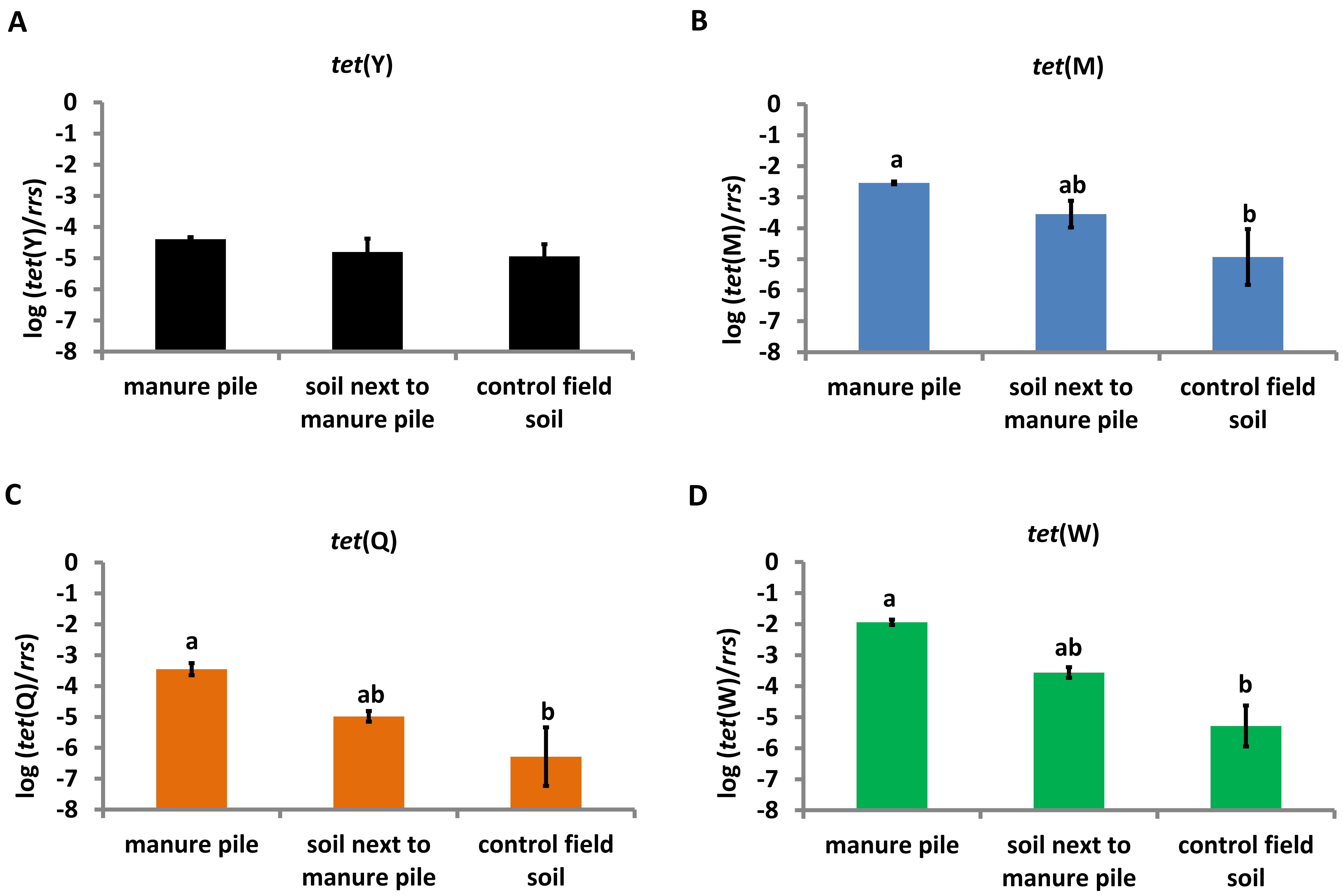

Supplement: Supplementary file 1 [file DataSheet1.PDF]
